# Understanding what citizens think about Antimicrobial Resistance: Deliberative Polling® in six middle-income countries

**DOI:** 10.12688/wellcomeopenres.24803.2

**Published:** 2026-05-14

**Authors:** Marc Mendelson, Alice Siu, Louise Gough, Hamish Morrow, James Fishkin, Sally Davies

**Affiliations:** 1 The Trinity Challenge, Master’s Lodge, Trinity College, Cambridge, CB2 1TQ, UK; 2 Division of Infectious Diseases and HIV Medicine, University of Cape Town Faculty of Health Sciences, Groote Schuur Hospital, Cape Town, South Africa; 3 Deliberative Democracy Lab, Stanford University Freeman Spogli Institute for International Studies, Stanford, California, CA 94305-6044, USA

**Keywords:** Antimicrobial resistance, AMR, deliberative polling, citizens, political declaration, policy development

## Abstract

**Background:**

The pandemic of antimicrobial resistance (AMR) will only be mitigated by policy action and innovation and importantly, supported by local and community action. With the United Nations General Assembly high level meeting on AMR taking place in September 2024, we decided to ascertain citizens’ understanding of the issues and prioritisation for action.

**Methods:**

From June to August 2024, while intergovernmental negotiations on the outcome document were taking place, we used Deliberative Polling ^®^, a methodology founded on deliberative democratic theory, in six middle income countries across three continents to explore people’s understanding and support for 45 policies that were likely to feature in the political declaration.

**Results:**

In total 2419 participants were randomised to deliberation intervention (written and video information, facilitated online small group discussions, and expert plenary sessions) or control groups who only completed the pre- and post-deliberation surveys. Support increased significantly through deliberation for 3/4 of the proposals (>90% for 2/3), as well as on knowledge about AMR and internal political efficacy. Proposals relating to infection prevention were most heavily supported across all six countries. We found regional variation in support for proposals relating to informal antibiotic access and the use of antibiotics in food production, with less support for selected proposals from South America.

**Conclusions:**

Deliberative polling is a powerful method of large scale community engagement and this is new for AMR helping us to understand the views of the public relating to policies that will require their support to enact.

## Plain Language Summary

**Understanding what citizens think about Antimicrobial Resistance: Deliberative Polling ^®^ in six middle-income countries**

In 2024, increasing resistance of bacteria to antibiotics, which impacts on our health, food, and socioeconomic security was debated at the United Nations General Assembly. This led to a political declaration, which included commitments for action that require public adoption to be implemented successfully.

In this study, researchers examined the level of support/opposition for these policies from citizens of 6 middle-income countries. They used Deliberative Polling ^®^, a method that assesses what a representative and informed sample would think about policy proposals after extended, evidence-based discussions.

There was a high level of collective support for the majority of proposals, which increased through the deliberative process. Antibiotic access and food security proposals showed regional variation in support. Deliberation also increased knowledge about antibiotic resistance and participants’ perception of their influence on public policy and ability to create changes in their government.

The importance of these findings is that there was high levels of public support for policies that could diminish antibiotic resistance in countries that suffer from a high burden of antibiotic-resistant bacterial infections, increasing the likelihood of their successful adoption. Polling countries with different socioeconomic resources would give a clearer picture of the global view.

## Introduction

Addressing global challenges requires worldwide support from leaders, politicians, and the private sector, yet such challenges are rarely resolved without pull from the general public. Increasing levels of antibiotic resistance in bacteria, (often referred to as antimicrobial resistance, AMR), is one such challenge impacting human health, our food chain, and the environment. It poses an existential threat to humanity by compromising health, food, environmental, and socioeconomic security. We set out to learn what proposals for action would the public support if they could consider the issue in depth? Also, is it possible to explore a complex public health challenge of this kind with the general public in low- and middle-income countries (LMICs)?

Bacterial infections cause approximately 8 million sepsis-related deaths/year. ^1^ Access to effective antibiotics is fundamental to survival from sepsis and to reducing risks from modern medical interventions such as surgery, cancer care, and transplantation. Antibiotics are also integral to reaching our sustainable development goals on child and maternal survival, and in supporting the health of vulnerable populations - newborns, elderly, people living with HIV and other immunosuppressive conditions. Similarly, the agrifood industry depends on antibiotics to maintain food production.

The impact of antibiotic resistance is daunting. Over the next 25 years, estimates suggest that 39 million people will die as a direct result of antibiotic-resistant bacterial infections. If we do not mitigate this pandemic, the deaths will outweigh those from HIV, tuberculosis, and malaria combined. ^2^ Annual livestock loss from antibiotic-resistant bacterial infections is estimated between $575 - $953 billion in cumulative global gross domestic product (GDP) over the same period, affecting the consumption needs of between 746 million and 2 billion people. ^3^ Estimates by the World Bank suggest that failure to mitigate AMR could wipe away 3.8% of global GDP each year by 2050, pushing 24 million people into poverty. ^4^ The effects of climate change on pathogens and their vectors is already accelerating bacterial resistance, through direct effects on bacteria themselves and by increasing the burden of infectious diseases in general e.g. increasing vector-borne infections by providing new ecologies for vectors. ^5, 6^ This in turn will increase transmission of pathogens to humans, resulting in increasing presentation of people with undifferentiated fevers to prescribers who will often inappropriately prescribe antibiotics.

Many interventions to mitigate AMR will require population-level behaviour change, which will have important trade-offs for the public, making it necessary to understand their level of support for the policies that will affect them. This study was designed to bring their perspectives systematically into the policy dialogue.

Several methodologies are already used to gauge public opinion. Standard polls are the easiest to perform, having the potential for good levels of representation. Furthermore they are relatively cheap. However, their accuracy can be affected by ‘phantom opinions’ i.e., people may answer at random rather than admit they do not know. ^7^ Bishop’s experiment in 1975, polled American citizens on a completely fictitious Act, the ‘Public Affairs Act of 1975’, and found that 20–40% of Americans were willing to offer opinions on a law that did not exist. ^8^ The experiment was replicated by YouGov, during the Obama presidency, suggesting that he would repeal the Act. Republicans were over 10 times more likely to disagree with the president on this issue than Democrats. ^9^

Smaller-scale consultation methods such as responsive dialogues, ^10^ citizen juries, ^11^ and citizen assemblies ^12^ allow more in-depth deliberation, but are unrepresentative at a population level. A disadvantage of juries and their larger, citizen assembly counterparts, is that they work to reach consensus, which may pressure participants to agree on a position or ‘verdict’.

Deliberative Polling ^®^, a methodology founded on deliberative democratic theory allows for a much larger, representative cohort of participants. ^13^ Members of the public are led through a process of deliberation to assess what they would think under ‘ *good*’ conditions i.e., having access to accurate, evidence-based considerations, weighing for and against the proposed policy alternatives or proposed actions. In addition, participants have an opportunity to get their questions answered by balanced panels of competing experts representing different points of view. At the end of one or two days of deliberation, their final considered judgments are gathered in confidential questionnaires, identical to those they completed pre-deliberation. The before and after deliberation opinions can then be contrasted with opinions of the control groups, who take the same questionnaires. Deliberative Polling has been used in over 150 studies in over 50 countries. ^13^

We undertook a randomised Deliberative Poll to determine the level of support from members of the public in six middle-income countries (MICs) in Africa, Asia, and South America for AMR policy proposals that were either likely to be part of, or closely aligned to, the Political Declaration of the United Nations General Assembly (UNGA) High-Level meeting on Antimicrobial Resistance (HLM-AMR) in September 2024. ^14^ The proposals could fundamentally affect participants and would require public support for implementation. We believe that this project offers proof of concept that such public consultations are not only possible but also relevant to policy-making in relation to AMR.

## Methods

The project employed Deliberative Polling ^®^ methodology to assess attitudes towards antimicrobial resistance in six MICs: Brazil, Colombia, Nigeria, Tanzania, Indonesia, and India. These six MICs were selected as they have high rates of antibiotic resistance ^2^ and the Stanford University’s Deliberative Democracy Lab had previous experience of conducting Deliberative Polls in these countries. A comparison of country-level demographics and AMR rates is depicted in Table 1. The Deliberative Democracy Lab received ethics approval from the Stanford University Institutional Review Board. The Stanford IRB protocol number for this study was 35343. This study was conducted in accordance with the ethical standards of Stanford University’s Institutional Review Board on 14th June 2024. Informed written consent was obtained from all individual participants included in the study. All aggregate and published data were anonymized to protect participant confidentiality and such anonymity does not distort or alter its scientific meaning.

**Table 1.** A comparison of country-level demographics and AMR rates.

Measurement	Brazil	Colombia	Nigeria	Tanzania	India	Indonesia
Income level	Upper-Middle	Upper-Middle	Lower-Middle	Lower-Middle	Upper-Middle	Upper-Middle
Population ∞, 2026	21,35,62,666	5,39,36,226	24,24,31,832	7,17,88,597	1,46,76,25,576	28,70,95,825
Official Language(s)	Portuguese	Spanish	English	Swahili; English	Hindi; English	Indonesian
GDP per Capita ∞ ($)	10,578	8,249	1,200	1,302	2,818	5,074
Gini Coefficient §	51.6	53.9	35.1	40.5	25.5	34.9
Multidimensional Poverty Index * (MPI)	0.016 (2015)	0.020 (2015)	0.175 (2021)	0.221 (2022)	0.069 (2019/2021)	0.014 (2017)
MPI Intensity of Deprivation ** (%)	42.5	40.6	52.9	46.9	42	38.7
AMR ¢ Sepsis deaths Bacteria-attributed AMR-associated AMR-attributable	135,407 30,341 19,597 4,855	741,963 199,075 129,826 31,662	106,000,000 360,645 226,859 50,516	236,128 70,251 42,196 9231	433,000,000 158,000,000 987,254 266,734	683,880 298,063 146,531 36,508
National Action Plan for AMR ¶	Yes, 2018	Yes, 2022	Yes, 2024	Yes, 2022	Yes, 2025	Yes, 2022

* An MPI value is the product of the incidence (H, or the proportion of people who live in multidimensional poverty) and intensity of poverty (A, or the average deprivation score among multidimensionally poor people). MPI values range from 0 to 1, and higher values imply higher poverty https://hdr.undp.org/content/2025-global-multidimensional-poverty-index-mpi#/indicies/MPI

** The measure of the average proportion of weighted indicators in which people identified as multidimensionally poor are deprived. It essentially quantifies the "depth" of poverty by calculating the average share of deprivations poor households experience simultaneously

^¢^ Institute of Health Metrics, 2021. https://vizhub.healthdata.org/microbe/

^∞^ Worldometer. https://www.worldometers.info/world-population/population-by-country/

^§^ Gini Coefficient (Gini Index or Gini ratio) is a measure of income distribution. The higher the coefficient, the greater the gap between incomes of a country’s richest and poorest people. https://worldpopulationreview.com/country-rankings/gini-coefficient-by-country

^¶^ WHO Library of National Action Plans https://www.who.int/teams/surveillance-prevention-control-AMR/national-action-plan-monitoring-evaluation/library-of-national-action-plans

The Deliberative Polling ^®^ intervention includes background materials, small group deliberations, and plenary sessions with expert panel question and answer sessions. The process measures both participants’ final opinions on each proposal, as well as shifts in opinion resulting from their informed deliberations. We aimed to recruit 400 citizens per country, randomised equally into intervention and control groups. The sample size balanced the ability to recruit a stratified random sample of the country’s adult population and our pre-determined budget.

Recruitment was conducted in country by YouGov, Random Dynamic Resources (RDR), and Alvara YouGov (India, Brazil, and Colombia) is a global public opinion company that operates online panels in all regions of the world. In many countries, many of their panels have millions of panellists, which allow for greater ability to access a representative population sample. YouGov panels are opt-in and use proprietary weighting algorithms to ensure accurate representation. YouGov recruited the nationally representative samples for India, Brazil, and Colombia. RDR (Nigeria and Tanzania) is a leading survey organization in Sub-Saharan Africa and the Middle-East and North Africa. RDR employed address-based probability sampling in-person and by phone. Knowing the target population and the level of commitment required for this event, RRDR chose these methods to ensure a high turnout, even though they are more time and resource intensive. Alvara (Indonesia) is a research and consulting company that specializes in conducting online surveys using probability-based sampling.

To ensure the samples in each country were representative of the general population, they used accurate and high resolution population data such as census data to determine benchmarks for critical demographic variables (gender, age, education level, and domicile), which may differ by country. The intervention and control group participants received between US$1 and $5 dollars, depending on the country, to complete the pre- and post-deliberation survey. In addition, the intervention group received a total amount between $75 and $100 inclusive of the pre- and post-survey incentives, as a compensation for their time and effort. During the recruitment process, participants are asked whether they would need additional funds to ensure their participation. Examples include funds for childcare, dependent care, boosting of internet quality, and/or funds for equipment, such as earphones and/or mobile devices. The polling firms work with each participant to ensure they have the necessary additional funds to ensure they are able to participate in the event if they wish to do so.

**Figure 1.**
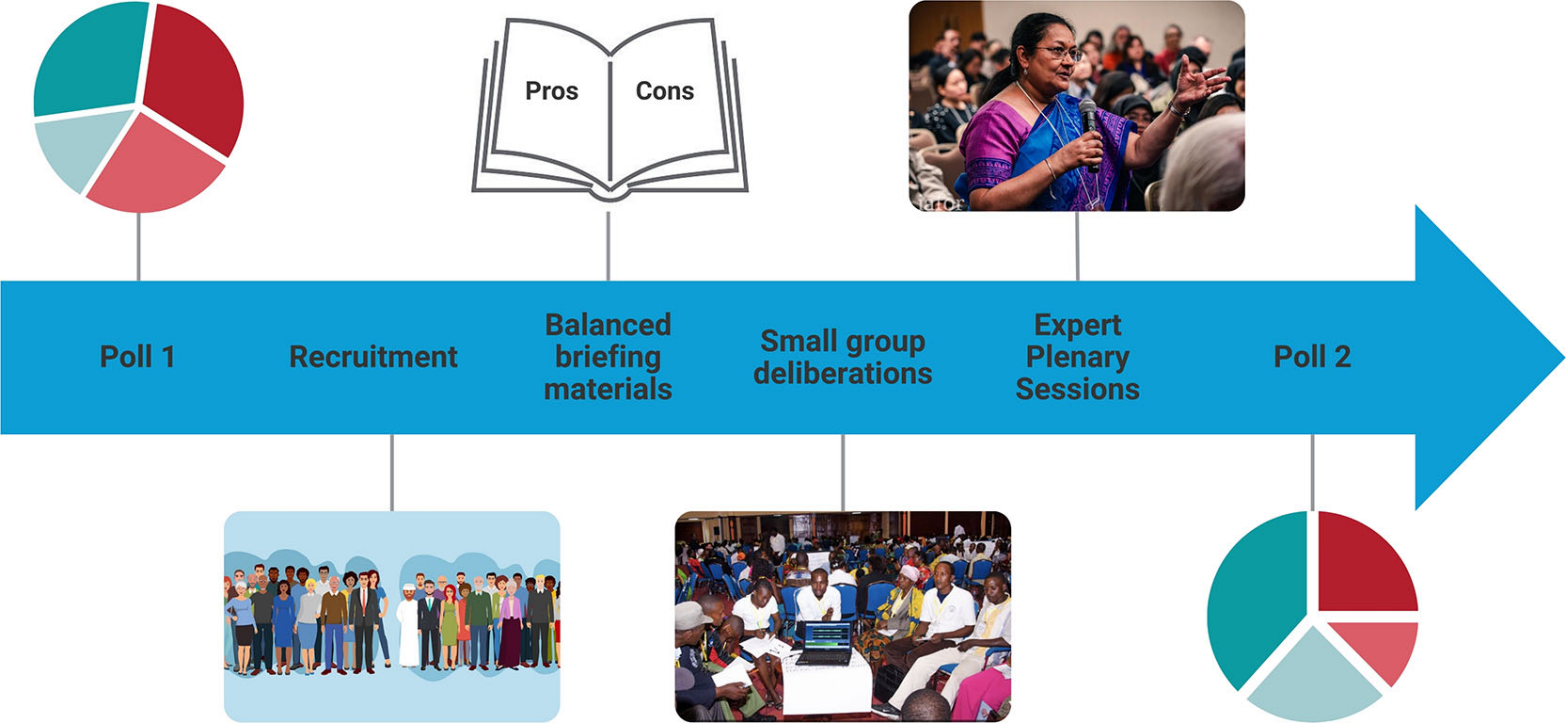
The Deliberative Polling ^®^ process.

The methodology began with a pre-deliberation survey of all participants. After completion of the pre-deliberation survey, participants were randomly assigned to either the deliberation intervention or control groups. Whilst participants in the intervention group were then invited to participate in the deliberation process, control group participants were only required to complete the post-deliberation survey, and nothing else ( Figure 1).

## Deliberation

The background materials (both written and video) in English, Hindi, Indonesian, Portuguese, and Spanish provided information on proposals including pros and cons to each, highlighting trade-offs for consideration. Briefing materials were reviewed by a Trinity Challenge Expert Advisory Group comprising AMR and policy experts.

In each country, intervention groups participated in small group discussions (75 minutes) and plenary sessions (75 minutes) for each set of proposals over a two-day period. The entire intervention was approximately 10 hours. Small group discussions were conducted on the Artificial Intelligence (AI)-assisted Stanford University Online Deliberation Platform. ^15^ The platform moderated video-based discussions, controlled the queue for talking, nudged those who had not volunteered to talk, intervened if there was incivility, and moved the group of 8–10 deliberants through the agenda of policy proposals and their pros and cons. Near the end of each discussion, it prompted groups in formulating two key questions to pose to the expert panels. Online plenary sessions had a minimum of two experts fielding the groups’ questions, with a human moderator. After deliberation, both the intervention and control groups completed the final (post-deliberation) survey.

## Surveys and proposals

The same survey was used at both timepoints. It included questions on demography – gender, age, domicile (urban, suburban, rural), and level of education, grouped as: primary - high school completion or below; secondary; post-secondary (including non-tertiary education and short-cycle tertiary education typically equating to vocational or trade school); and tertiary - bachelor’s degree or equivalent and higher.

Questions included sections on knowledge about AMR and on political efficacy, especially internal political efficacy (“I have opinions worth listening to”). Participants were asked to score their level of support/opposition for each proposal on a Likert Scale from 0–10 i.e., strongly oppose to strongly support.

Participants in the intervention arm were asked to deliberate on 45 proposals ( Table 2) which were chosen for their likelihood of being incorporated in, or aligned to, the UNGA HLM-AMR political declaration being negotiated in 2024. Six categories of proposals were chosen - equity, access, and stewardship (14 proposals), infection prevention (11), food security (6), targets (5), surveillance (5), and awareness and education (4).

**Table 2.** 45 proposals used in the deliberation.

**Equity, Access and Stewardship**
1. Governments in low- and middle-income countries should ensure appropriate accessed to antibiotics for all citizens including those who have difficulty in accessing the health system 2. Governments in low- and middle-income countries should increase access to antibiotics to support all farmers for their animals, including those who have little access to veterinarians 3. Governments should develop clear guidelines and support for controlled informal dispensing and monitoring of the most widely needed basic oral antibiotics without a doctor’s or veterinarian’s prescription 4. Registered pharmacists should be able to dispense basic oral antibiotics over the counter for some community-acquired infections without a prescription 5. Informal antibiotic sellers such as Patent and Proprietary Medicine Vendors and informal drug shops should be regulated and monitored to dispense a limited number of basic oral antibiotics for specific infections under strict conditions 6. Veterinarian assistants or allied veterinary professionals should be allowed to dispense antibiotics to farmers for a limited number of infections without a prescription 7. Governments should develop monitoring (and evaluation) mechanisms to include informal sales of antibiotics 8. Governments should report the volumes of sales from these informal sources annually to the Global Antibiotic Surveillance System, a global monitoring system set up by the World Health Organization 9. Governments should incentivise informal sellers to use only quality-assured antibiotics 10. An international initiative is needed to ensure affordable steady supply of quality-assured basic oral antibiotics in low- and middle-income countries including local manufacturing 11. An international initiative is needed to ensure availability of proven/regulated point of care diagnostic tests to support appropriate antibiotic use in all countries. 12. Governments should make currently available validated point of care diagnostic tests available at every primary health outlet 13. Point of care rapid diagnostic tests need to be available and part of care pathways, and should be reimbursed by the State of Medical Insurance Companies 14. There should be national certification standards from manufacture of antibiotics in all countries for safe waste/outflow disposal, with penalties if standards are not met.
**Infection Prevention**
15. National governments should prioritise infection prevention tools (clean water, safe sanitation and vaccination) to reduce the burden of infectious diseases 16. Everyone should have access to clean water and safe sanitation (WASH) in their community and health system. 17. Specific government funding should go to healthcare facilities to ensure that all have access to clean water for hand hygiene 18. Immunization for WHO-recommended childhood vaccination should be scaled up towards universal/full coverage to capture all children 19. An adult programme of vaccination should be created by national governments to ensure vaccination of vulnerable populations for influenza and pneumonia 20. Influenza vaccination should be incentivised for patient-facing healthcare workers 21. Hospital accreditation should be linked to infection prevention, such as using hand washing rates as a factor 22. Hospital should be mandated to publish their hand hygiene rates annually 23. Farms should ensure good infection prevention and biosecurity 24. Farming, including fish, should use vaccination 25. Farm workers should be taught to wash their hands
**Food Security**
26. An identifying label should accompany food that is produced without the use of routine antibiotics 27. Large-scale food suppliers – supermarkets, restaurant chains, intensive farming etc., - should be incentivised to promote the use of food produced without routine antibiotics 28. Large-scale food suppliers - supermarkets, restaurant chains, intensive farming etc., - should be regulated to promote the use of food produced without routine antibiotics 29. Governments should regulate farmers to establish infection prevention programs on farms (including vaccination) linked to an inspection program 30. Group prevention therapy with antibiotics for sick animals should be under the management of a veterinarians or allied professional 31. Governments should ban from food production systems, the use of antibiotics that are critically important for humans
**Awareness and Education**
32. Food produced with the routine use of antibiotics should be identified by an international standard such as a hazard-warning symbol or label 33. Hand hygiene and infection preventions should be part of the standard curriculum in schools from the start of schooling through to graduation 34. Awareness campaigns should focus on the risks and harms of taking unnecessary antibiotics on humans, food security, and the environment 35. National governments should increase funding for awareness campaigns into vaccine hesitancy and improving understanding about people’s behaviours relating to vaccination
**Targets**
36. There should be global targets to address bacterial resistance to antibiotics, just like global targets for climate 37. There should be a global target to reduce human deaths from antibiotic resistance 38. There should be a global target to stop the use of medically critical antibiotics in the food chain 39. There should be a global target to stop the use of antibiotics for growth promotion and infections prevention without veterinary advice 40. Antibiotic resistance should be included as a cause of death on all national death certificates to enable collection of data on mortality
**Surveillance**
41. National surveillance programs should be in place to monitor infection rates, bacterial types, antibiotic use, and antibiotic resistance in humans, animals (including fish) and water systems 42. International funding support should be available for surveillance in countries willing to invest in laboratory capacity, monitoring, and reporting and where systems are not yet available. 43. Surveillance data should be reported nationally, regionally, and to the World Health Organization, Food and Agriculture Organisation, and the World Organisation for Animal Health 44. Laboratory systems for measuring antibiotic resistance in bacteria for humans and animals should be integrated with shared One Health resources 45. Data from point-of-care rapid diagnostic tests should be incorporated into routine national surveillance reporting.

## Analysis

Quantitative analysis of demographic and attitudinal representativeness of the sample, opinion change of policy attitudes before and after deliberation, and the effects of explanatory variables on opinion change was conducted using Python. These included empirical premises, which relate to consequences, as well as knowledge and value questions. Mean values on the Likert scale (0–10) and percentage support for each proposal in each country group were calculated and statistical significance was assessed using the paired sample t-test. We included systematic comparison of changes in the intervention and control groups, assessing whether participants changed their opinions and, if so, why. We further examined how demographic variables played a role in any changes or lack of changes in opinion.

For qualitative analysis of the transcripts, we used the Deliberation Analyzer, a proprietary text-analysis platform. The Deliberation Analyzer is a joint effort between Stanford University’s Deliberative Democracy Lab and File read, designed as a specialized natural language processing pipeline for analysing deliberations. It utilizes two language models: BERT and GPT-4. At the time of analysis for this paper, BERT-based models were still among the most advanced for language analysis, particularly excelling in tasks like emotional analysis, where it identifies strong emotions in text. While OpenAI’s GPT models have gained widespread recognition for their versatility, BERT remains superior for analytical tasks. However, GPT-4, at the time, was undoubtedly the best generative language model, excelling in areas like summarization and storytelling. To maximize effectiveness, Fileread employs BERT for deliberation analysis, with the results then processed through GPT-4 for interpretation and summarization.

Transcripts from each small group were analysed to determine what arguments were made in discussions, whether and how often pro and con arguments were made towards specific proposals, and to provide summaries of the discussions, at group level and for the entire event. Summaries provided a basic understanding of reasoning during deliberations and types of arguments used, which will be the subject of a separate paper. While these transcripts will be largely reported separately, selected quotes from the groups to highlight participants’ opinions are presented here.

## Results

There were a total of 2,419 participants, 1,280 in the intervention groups and 1,139 controls across six countries. Recruitment targets were met, albeit with non-significant variation between countries ( Table 3). A higher number of invitations to the deliberations were extended than those that completed the deliberative process. This is expected as the deliberative event neared some participants cancelled due to logistical conflicts, for example. The percentage of attendance, in these countries, from the initial invitation are comparable to previous deliberations carried out by the Stanford Deliberative Democracy Lab. There are substantial differences in the attendance rate between Brazil, Colombia, and India in comparison to Indonesia, Nigeria, and Tanzania because Brazil, Colombia, and India were recruited through YouGov, from their online opt-in panels ( Table 4). Participants on opt-in panels are often harder to reach, therefore more invitations are sent out to ensure participation. In contrast, Indonesia, Nigeria, and Tanzania were carried out with local polling firms with higher-touch recruitment methodologies including phone and text with participants. Therefore, the polling firms were able to extend fewer invitations and achieve a higher attendance rate.

**Table 3.** Number of participants in intervention and control groups by country.

Country	Treatment Group	Control Group
Tanzania	185	206
Nigeria	203	210
India	231	160
Brazil	188	178
Colombia	275	185
Indonesia	198	200

**Table 4.** Attendance rate of participants in the intervention deliberation groups as a percentage of those invited.

Country	Invited to Deliberation	Participated in Deliberation	Attendance % from invitation
Tanzania	221	185	84%
Nigeria	221	203	92%
India	700	231	33%
Colombia	935	275	29%
Brazil	812	188	23%
Indonesia	238	198	83%

Demographic variables are shown in Table 5. Participants from all countries were well matched for gender, apart from India, where two-thirds were male. Collectively, the largest percentage of participants were 25–34 years old. India had the largest proportion of the youngest participants i.e., 18–24-year-olds, and Tanzanian participants had the youngest overall cohort, all <44 years old. Urban or suburban domiciles predominated in all countries, although less in Brazil and Colombia (61–75%) than the other four countries (88.7% – 97.6%). Overall, secondary education or lower was the highest level of education attained by participants, with one third of Brazilian participants having had primary level education or less, contrasting to three quarters of Nigerians who had attained tertiary level education or higher. Comparisons along several demographic characteristics by country revealed that control and intervention groups were well balanced for age and gender. Indonesia and Nigeria’s intervention groups tended towards a higher level of education than their control counterparts, while India and Tanzania’s intervention groups were more likely to reside in urban settings compared to controls.

**Table 5.** Demographics – Control and Intervention groups. Results are expressed in % proportion in each variable in each country.

Characteristic	Group	Variable	Tanzania	Nigeria	India	Colombia	Brazil	Indonesia
Gender	Control	Male (%)	51.8	51.2	69.4	52.4	49.4	50.5
		Female (%)	48.2	48.8	30.6	47.6	50.6	49.5
	Intervention	Male (%)	47.6	51.4	67.5	53.5	48.7	50.6
		Female (%)	52.4	48.6	32.5	46.6	51.3	49.4
Age, years	Control	18-24 (%)	21.4	19.5	32.3	17.3	18.0	20.5
		25-34 (%)	31.8	27.3	33.5	29.2	25.3	32.0
		35-44 (%)	13.5	23.4	18.4	19.5	22.5	23.0
		45-54 (%)	6.8	17.6	10.1	20.0	16.3	22.0
		55+ (%)	26.6	12.2	5.7	14.1	18.0	2.5
	Intervention	18-24 (%)	17.2	20.5	32.0	16.0	16.4	18.2
		25-34 (%)	31.1	37.5	32.9	29.8	26.5	33.8
		35-44 (%)	31.1	18.0	19.5	21.1	21.2	24.8
		45-54 (%)	20.6	8.5	10.0	17.8	17.5	22.7
		55+ (%)	0.0	15.5	5.6	15.3	18.5	0.5
Education	Control	Primary or less (%)	25.1	0.0	7.1	5.3	37.5	0.0
		Secondary (%)	41.9	24.0	63.3	59.0	48.4	59.3
		Post-Secondary * (%)	22.3	28.2	6.3	0.0	4.1	0.0
		Tertiary or higher(%)	10.6	47.8	23.3	35.7	10.0	40.7
	Intervention	Primary or less (%)	17.4	0.0	6.3	5.8	33.2	1.0
		Secondary (%)	47.9	7.0	64.4	58.6	51.5	33.7
		Post-Secondary * (%)	23.4	18.8	6.3	0.0	4.8	0.0
		Tertiary or higher(%)	11.3	74.2	23.1	35.6	10.6	65.3
Urbanicity	Control	Urban or Suburban	79.0	99.6	77.0	61.4	75.5	95.4
		Town, rural or other	21.0	0.4	23.0	38.6	24.5	4.6
	Intervention	Urban or Suburban	95.9	97.8	88.7	63.6	69.2	95.0
		Town, rural or other	4.1	2.2	11.3	36.4	30.9	5.0

^*^ Post-secondary = post secondary non-tertiary or vocational education.

Variation in support/opposition for proposals in relation to gender of participants is shown in Figure 2a and Figure 2b. Gender was only a significant factor in the average proposal ratings for Brazilian women, who supported 8/45 proposals significantly higher (average 1.01 on the Likert scale) than men after deliberation across different proposal categories. The eight proposals were: 1) Registered pharmacists should be able to dispense basic oral antibiotics over the counter for some community-acquired infections without a prescription, 2) Large-scale food suppliers – supermarkets, restaurant chains, intensive farming etc., – should be **incentivized** to promote the use of food produced without routine antibiotics, 3) Large-scale food suppliers – supermarkets, restaurant chains, intensive farming etc., – should be **regulated** to promote the use of food produced without routine antibiotics, 4) Governments should ban from food production systems the use of antibiotics that are critically important for humans, 5) Laboratory systems for measuring antibiotic resistance in bacteria for humans and animals should be integrated with shared One Health resources, 6) Farming, including fish, should use vaccination, 7) Governments should regulate farmers to establish infection prevention programs on farms (including vaccination) linked to an inspection program, and 8) There should be a global target to stop the use of medically critical antibiotics in the food chain. There is not an obvious trend for these several proposals that were more highly supported by Brazilian women after deliberations. All countries except Nigeria and Indonesia showed an incremental increase in support for specific proposals with increasing age after deliberation. Tanzanian participants had the largest significant increases (0.033 to 0.093 mean points increase for each additional year of age for 15/45 proposals), Brazilians (0.017 to 0.03 for 7/45 proposals including all four dealing with antibiotic stewardship), Colombians (0.021 – 0.035 for 5/45 proposals, including three related to target setting), and Indians (0.016–0.031 4/45, including 3/4 awareness and education proposals).

**Figure 2a.**
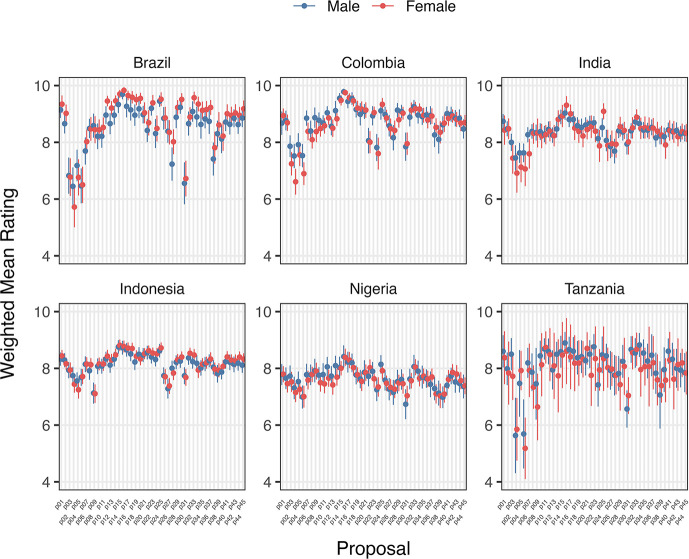
Weighted Mean of Proposal Rating by Gender before deliberation (T1).

**Figure 2b.**
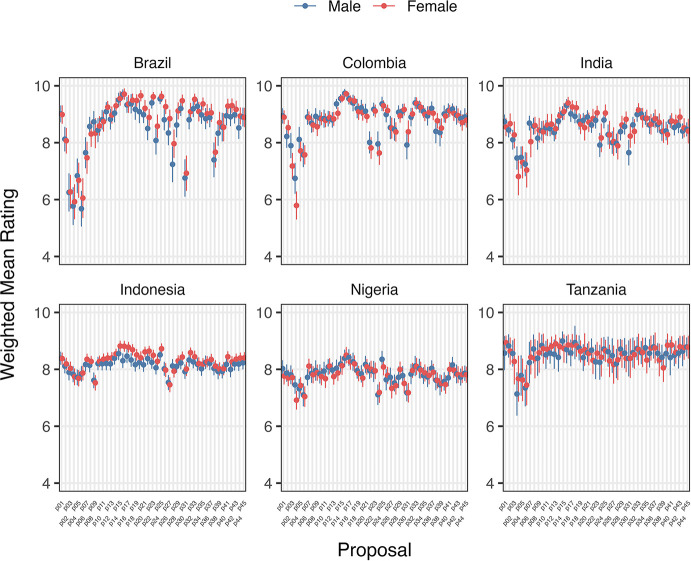
Weighted Mean of Proposal Rating by Gender after deliberation (T2).

**Figure 2c.**
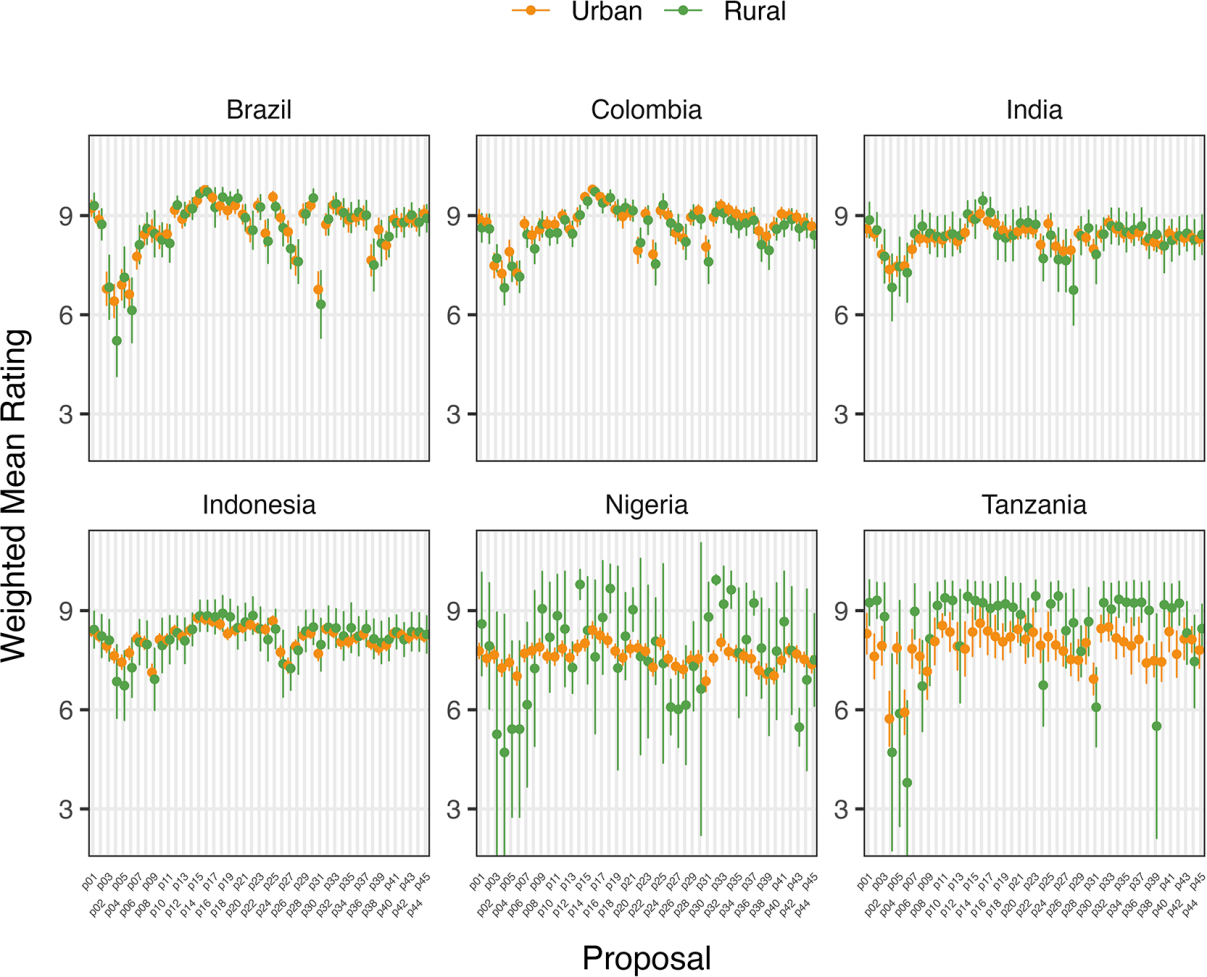
Weighted Mean of Proposal Rating by Urbanicity before deliberation (T1).

Place of domicile was not a statistically significant factor in determining mean ratings of proposals for Brazilian, Colombian, or Indonesian participants before or after deliberation ( Figure 2c and Figure 2d). Rural participants in India rated 7/45 proposals higher (0.67 to 1.12 points) and in Tanzania, two proposals lower (-1.76 to -2.6 points) on average after deliberation than urban participants. Neither demonstrated any clear trend in type of proposals rated differently. However, Nigeria’s rural participants rated 11 proposals higher than urban counterparts after deliberation, including those relating to regulation of food suppliers to promote the use of food produced without routine antibiotics. Except for Tanzanian participants, higher levels of education were generally associated with increased rating of proposals after deliberation.

**Figure 2d.**
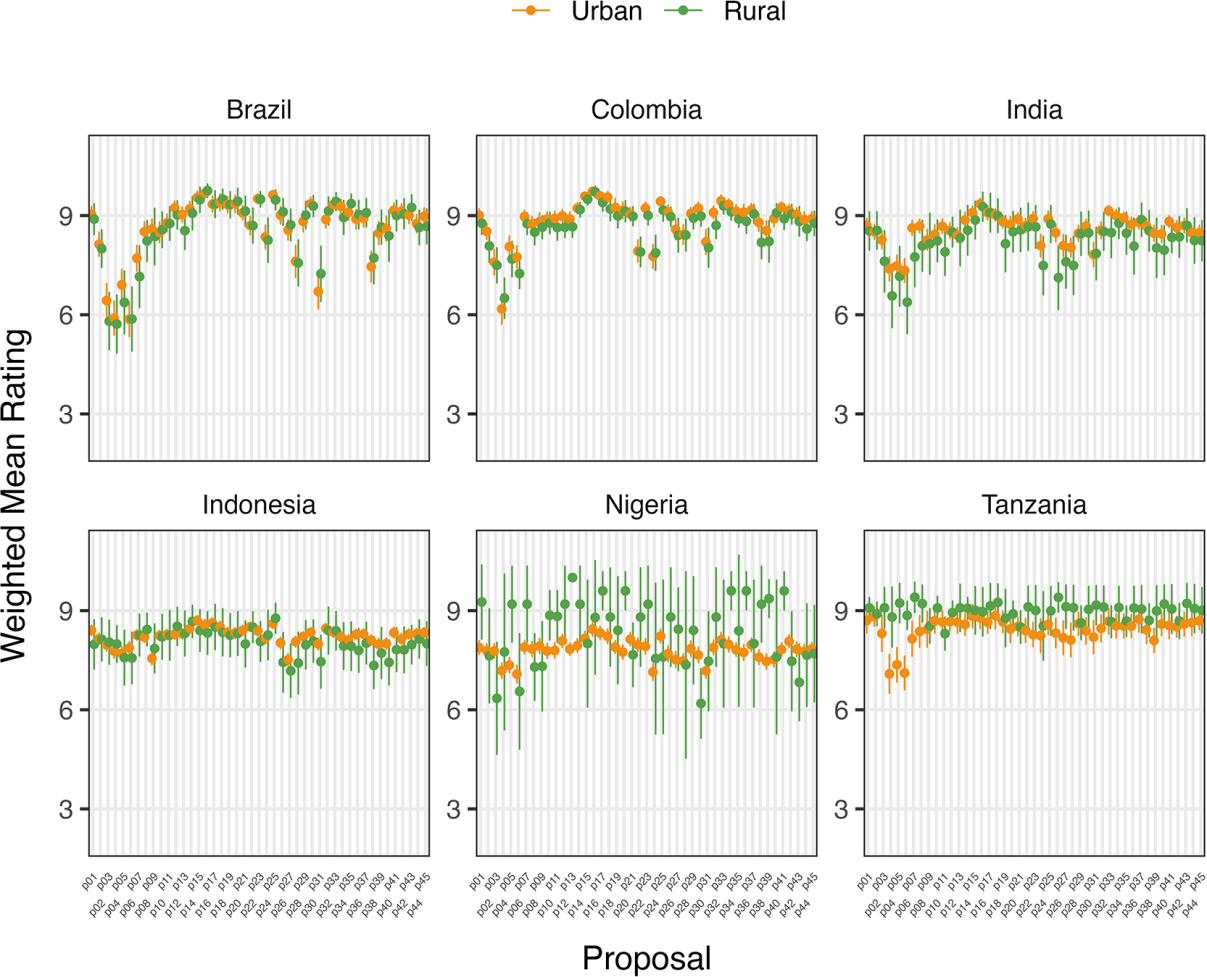
Weighted Mean of Proposal Rating by Urbanicity after deliberation (T2).

## Top ten proposals after deliberation

There are two ways of thinking about the results of Deliberative Polls. First, what are the final considered judgments of the intervention group? These may, or may not be different from where they started. If they are similar, they will have survived the test of adverse argument considering the pros and cons for each proposal. Second, what are the final considered judgments and the opinion changes as in some cases they will be significantly different.

In terms of the final considered judgments about what needs to be done, the proposals relating to infection prevention were most heavily supported, with each country having 2–7 of the 11 infection prevention proposals among their most popular top 10 ( Table 6 and Table 7). Awareness and education proposals were second most popular across all countries.

**Table 6.** Proposals entering the top 10 after deliberation by topic and country.

Topic	Tanzania	Nigeria	India	Colombia	Brazil	Indonesia
Infection Prevention	2	2	6	5	7	7
Awareness & Education	1	1	3	2	2	2
Surveillance & Monitoring	3	3	0	0	0	0
Targets for Antibiotics	1	1	0	1	0	0
Stewardship	0	0	1	1	0	1
Access to antibiotics	3	2	0	0	0	0
Food Security	0	1	0	1	1	0

**Table 7.** Top 10 proposals selected by country.

Rank	Topic	Proposal
**Tanzania**		
1	Target for antibiotics	There should be a global target to stop the use of medically critical antibiotics in the food chain.
2	Access to antibiotics	Governments in low- and middle-income countries should increase access to antibiotics to support all farmers for their animals, including those who have little access to veterinarians.
3	Access to antibiotics	Governments should develop clear guidelines and support for controlled informal dispensing and monitoring the most widely needed basic oral antibiotics without a doctor’s or veterinarian’s prescription
4	Surveillance and monitoring	National surveillance programs should be in place to monitor infection rates, bacterial types, antibiotic use, and antibiotic resistance in humans, animals (including fish) and water systems
5	Infection Prevention	Specific government funding should go to all healthcare facilities to ensure that all have access to clean water for hand hygiene
6	Infection Prevention	Immunization for WHO-recommended childhood vaccination should be scaled up towards universal/full coverage to capture all children
7	Access to antibiotics	Informal antibiotic sellers such as Patent and Proprietary Medicine Vendors and informal drug shops should be regulated and monitored to dispense a limited number of basic oral antibiotics for specific infections under strict guidelines but still without a prescription
8	Surveillance and monitoring	Data from point of care rapid diagnostic tests should be incorporated into routine national surveillance reporting
9	Awareness and education	Awareness campaigns should focus on the risks and harms of taking unnecessary antibiotics on humans, food security, and the environment
10	Surveillance and monitoring	Laboratory systems for measuring antibiotic resistance in bacteria for humans and animals should be integrated with shared One Health resources
**Nigeria**		
1	Awareness and education	Hand hygiene and infection preventions should be part of the standard curriculum in schools from the start of schooling through graduation
2	Targets for antibiotics	There should be a global target to reduce human deaths from antibiotic resistance
3	Surveillance and monitoring	Data from point of care rapid diagnostic tests should be incorporated into routine national surveillance reporting
4	Infection Prevention	Farms should ensure good infection prevention and biosecurity
5	Surveillance and monitoring	National surveillance programs should be in place to monitor infection rates, bacterial types, antibiotic use, and antibiotic resistance in humans, animals (including fish) and water systems
6	Access to antibiotics	Informal antibiotic sellers such as Patent and Proprietary Medicine Vendors and informal drug shops should be regulated and monitored to dispense a limited number of basic oral antibiotics for specific infections under strict guidelines but still without a prescription
7	Infection Prevention	Hospitals should be mandated to publish their hand hygiene rates annually
8	Food security	Large-scale food producers – supermarkets, restaurant chains, intensive farming etc., - should be incentivized to promote the use of food produced without routine antibiotics.
9	Access to antibiotics	Governments should develop monitoring and evaluation mechanisms to include the informal sales of antibiotics
10	Surveillance and monitoring	Surveillance data should be reported nationally, regionally, and to the World health Organization, Food and Agriculture Organisation, and the World Organisation for Animal Health
** India**		
1	Infection Prevention	Everyone should have access to clean water and safe sanitation (WASH) in their community and health system
2	Awareness and education	Hand hygiene and infection preventions should be part of the standard curriculum in schools from the start of schooling through graduation
3	Infection Prevention	National governments should prioritise infection preventions tools (clean water, safe sanitation and vaccination) to reduce the burden of infectious diseases
4	Infection Prevention	Specific government funding should go to healthcare facilities to ensure that all have access to clean water for hand hygiene
5	Infection Prevention	Immunization for WHO-recommended childhood vaccination should be scaled up towards universal/full coverage to capture all children
6	Awareness and education	Awareness campaigns should focus on the risks and harms of taking unnecessary antibiotics on humans, food security, and the environment
7	Awareness and education	National governments should increase funding for awareness campaigns into vaccine hesitancy and improving understanding about people’s behaviours relating to vaccination
8	Infection Prevention	Farms should ensure good infection prevention and biosecurity
9	Infection Prevention	Hospital accreditation should be linked to infection prevention, such as using hand washing rates as a factor
10	Making best use of antibiotics	There should be national certification standards from the manufacture of antibiotics in call countries for safe waste/outflow disposal, with penalties if standards are not met.
**Colombia**		
1	Infection Prevention	Everyone should have access to clean water and safe sanitation (WASH) in their community and health system
2	Infection Prevention	National governments should prioritise infection preventions tools (clean water, safe sanitation and vaccination) to reduce the burden of infectious diseases
3	Infection Prevention	Specific government funding should go to healthcare facilities to ensure that all have access to clean water for hand hygiene
4	Awareness and education	Hand hygiene and infection preventions should be part of the standard curriculum in schools from the start of schooling through graduation
5	Infection Prevention	Immunization for WHO-recommended childhood vaccination should be scaled up towards universal/full coverage to capture all children
6	Awareness and education	Awareness campaigns should focus on the risks and harms of taking unnecessary antibiotics on humans, food security, and the environment
7	Infection Prevention	Farm workers should be taught hand hygiene
8	Making best use of antibiotics	There should be national certification standards from the manufacture of antibiotics in call countries for safe waste/outflow disposal, with penalties if standards are not met.
9	Targets for antibiotics	There should be a global target to reduce human deaths from antibiotic resistance
10	Food security	Group prevention therapy with antibiotics for sick animals should be under the management of a veterinarian or allied professional
**Brazil**		
1	Infection Prevention	Farm workers should be taught hand hygiene
2	Infection Prevention	Everyone should have access to clean water and safe sanitation (WASH) in their community and health system
3	Infection Prevention	Farms should ensure good infection prevention and biosecurity
4	Infection Prevention	National governments should prioritise infection preventions tools (clean water, safe sanitation and vaccination) to reduce the burden of infectious diseases
5	Infection Prevention	Influenza vaccination should be prioritised for patient-facing healthcare workers
6	Infection Prevention	Immunization for WHO-recommended childhood vaccination should be scaled up towards universal/full coverage to capture all children
7	Infection Prevention	An adult programme of vaccination should be created by national governments to ensure vaccination of vulnerable populations for influenza and pneumonia
8	Awareness and education	National governments should increase funding for awareness campaigns into vaccine hesitancy and improving understanding about people’s behaviours relating to vaccination
9	Awareness and education	Hand hygiene and infection preventions should be part of the standard curriculum in schools from the start of schooling through graduation
10	Food security	Group prevention therapy with antibiotics for sick animals should be under the management of a veterinarian or allied professional
**Indonesia**		
1	Infection Prevention	Farm workers should be taught hand hygiene
2	Infection Prevention	National governments should prioritise infection preventions tools (clean water, safe sanitation and vaccination) to reduce the burden of infectious diseases
3	Infection Prevention	Everyone should have access to clean water and safe sanitation (WASH) in their community and health system
4	Infection Prevention	Specific government funding should go to healthcare facilities to ensure that all have access to clean water for hand hygiene
5	Making best use of antibiotics	There should be national certification standards from the manufacture of antibiotics in call countries for safe waste/outflow disposal, with penalties if standards are not met.
6	Infection Prevention	Immunization for WHO-recommended childhood vaccination should be scaled up towards universal/full coverage to capture all children
7	Infection Prevention	Hospitals should be mandated to publish their hand hygiene rates annually
8	Awareness and education	Food produced with the routine use of antibiotics should be identified by an international standard, such as a hazard-warning symbol or label
9	Infection Prevention	Farms should ensure good infection prevention and biosecurity
10	Awareness and education	Hand hygiene and infection preventions should be part of the standard curriculum in schools from the start of schooling through graduation

## The effect of deliberation on support for proposals

Overall, the average level of support across countries after deliberation was over 70% and greater than 90% for two-thirds of proposals ( Table 8, Table 9, Table 10, Table 11, Table 12 and Table 13). In total, collective support for 43/45 proposals increased with deliberation, many with very high levels of support. Prioritisation of infection prevention through combined clean water and safe sanitation (WASH) and vaccination had an average support rating of 96.4% across MICs and ≥98% from Nigeria, India, and Colombia after deliberation. WASH-specific proposals alone, averaged 97% support post deliberation, with 100% of Nigerian participants supporting increased access to community-level and health system WASH. One participant opined that “ *The importance of clean water cannot be overemphasized*” and that “ *the role of the National government (in ensuring clean water access) should be emphasized.*” One Indonesian participant expressed that “ *the first national action must be to prepare infection prevention tools to reduce the burden of infectious diseases.*”

**Table 8.** Equity Access and Stewardship - mean ratings for each proposal, pre and post deliberation by country.

Proposal		Pre							Post							Difference						
	Measure	All	TZ	NG	ID	IN	CO	BR	All	TZ	NG	ID	IN	CO	BR	All	TZ	NG	ID	IN	CO	BR
Governments in low- and middle-income countries should ensure appropriate access to antibiotics for all citizens including those who have difficulty in accessing the health system.	Mean	8.51	7.77	8.04	8.21	8.69	8.87	9.37	8.76	8.81	8.40	8.25	8.84	9.09	9.06	0.25***	1.04***	0.36**	0.05	0.15	0.22*	-0.31
Governments in low- and middle-income countries should ensure appropriate access to antibiotics for all citizens including those who have difficulty in accessing the health system.	Oppose	2.6%	6.9%	1.7%	1.4%	2.3%	3.0%	0.3%	0.7%	0.0%	0.0%	2.2%	0.6%	0.0%	1.6%	-1.9%	-6.9%	-1.7%	0.8%	-1.7%	-3.0%	1.4%
	In the middle	6.2%	17.9%	1.6%	9.9%	4.8%	2.0%	3.6%	3.2%	3.5%	1.6%	7.5%	1.4%	2.1%	3.9%	-3.0%	-14.4%	-0.1%	-2.4%	-3.4%	0.1%	0.3%
	Support	88.3%	75.2%	96.7%	84.9%	89.5%	87.6%	95.0%	94.9%	96.5%	98.4%	88.6%	96.8%	95.4%	93.3%	6.6%	21.3%	1.7%	3.7%	7.3%	7.8%	-1.7%
	DK/NA	3.0%	0.0%	0.0%	3.8%	3.4%	7.4%	1.1%	1.2%	0.0%	0.0%	1.8%	1.2%	2.5%	1.2%	-1.8%	0.0%	0.0%	-2.1%	-2.2%	-4.9%	0.1%
Governments in low- and middle-income countries should increase access to antibiotics to support all farmers for their animals, including those who have little access to veterinarians.	Mean	8.25	7.25	7.84	8.04	8.65	8.61	8.95	8.34	8.88	8.12	8.12	8.63	8.25	8.00	0.09	1.63***	0.27	0.08	-0.02	-0.37**	-0.95***
	Oppose	4.8%	19.6%	3.5%	2.9%	1.7%	1.9%	1.9%	3.9%	0.8%	1.6%	3.2%	3.5%	6.1%	7.2%	-0.9%	-18.8%	-1.8%	0.3%	1.9%	4.2%	5.3%
	In the middle	6.3%	11.6%	3.6%	9.7%	4.1%	7.3%	1.8%	5.8%	4.5%	3.6%	8.6%	2.0%	6.3%	10.4%	-0.5%	-7.1%	0.0%	-1.2%	-2.0%	-1.0%	8.7%
	Support	85.4%	68.8%	93.0%	84.4%	92.4%	82.3%	90.5%	88.9%	94.7%	94.8%	86.5%	92.6%	84.4%	81.7%	3.5%	25.9%	1.8%	2.1%	0.2%	2.1%	-8.8%
	DK/NA	3.5%	0.0%	0.0%	2.9%	1.8%	8.6%	5.9%	1.4%	0.0%	0.0%	1.8%	1.8%	3.3%	0.7%	-2.1%	0.0%	0.0%	-1.2%	0.0%	-5.3%	-5.2%
Governments should develop clear guidelines and support for controlled informal dispensing and monitoring of the most widely needed basic oral antibiotics without a doctor’s or veterinarian’s prescription.	Mean	7.57	7.67	7.99	7.63	7.77	7.54	6.61	7.92	8.87	8.27	7.89	8.54	7.76	5.90	0.36***	1.20***	0.28	0.25	0.77***	0.22	-0.72*
	Oppose	9.8%	13.1%	3.7%	6.6%	4.3%	11.2%	21.2%	8.2%	0.6%	1.6%	4.0%	4.3%	10.2%	29.2%	-1.6%	-12.6%	-2.1%	-2.6%	0.0%	-1.0%	8.0%
	In the middle	9.2%	3.4%	3.9%	13.3%	12.0%	9.1%	12.6%	7.7%	1.8%	3.9%	11.5%	4.3%	10.7%	13.1%	-1.5%	-1.6%	0.0%	-1.8%	-7.7%	1.5%	0.5%
	Support	76.5%	83.4%	92.4%	77.2%	77.8%	72.6%	56.3%	82.2%	97.6%	94.5%	83.4%	89.5%	76.0%	53.3%	5.7%	14.3%	2.1%	6.2%	11.7%	3.4%	-3.1%
	DK/NA	4.5%	0.1%	0.0%	2.9%	5.9%	7.1%	9.8%	1.9%	0.0%	0.0%	1.1%	2.0%	3.2%	4.4%	-2.7%	-0.1%	0.0%	-1.8%	-4.0%	-3.9%	-5.4%
Registered pharmacists should be able to dispense basic oral antibiotics over the counter for some community-acquired infections without a prescription.	Mean	6.84	5.44	7.58	7.20	7.32	7.01	6.19	6.81	7.59	7.30	7.48	7.30	5.93	5.39	-0.03	2.15***	-0.28	0.28	-0.02	-1.08***	-0.80**
	Oppose	17.3%	34.2%	5.8%	9.9%	14.3%	16.0%	26.4%	17.0%	2.2%	7.2%	7.8%	13.9%	28.8%	37.6%	-0.3%	-31.9%	1.4%	-2.1%	-0.5%	12.8%	11.3%
	In the middle	12.4%	24.1%	7.0%	14.4%	8.0%	11.3%	11.5%	13.6%	25.3%	11.9%	9.5%	10.4%	12.5%	13.6%	1.2%	1.2%	4.9%	-4.9%	2.4%	1.3%	2.1%
	Support	67.0%	41.3%	87.2%	72.1%	72.2%	67.9%	57.1%	67.9%	72.5%	80.8%	81.5%	74.1%	54.6%	47.3%	0.9%	31.2%	-6.3%	9.4%	1.9%	-13.3%	-9.7%
	DK/NA	3.4%	0.5%	0.0%	3.5%	5.5%	4.8%	5.0%	1.6%	0.0%	0.0%	1.1%	1.6%	4.1%	1.4%	-1.8%	-0.5%	0.0%	-2.4%	-3.8%	-0.8%	-3.6%
Informal antibiotic sellers such as Patent and Proprietary Medicine Venders and informal drug shops should be regulated and monitored to dispense a limited number of basic oral antibiotics for specific infections under strict guidelines but still without a prescription.	Mean	7.44	7.61	7.74	6.90	7.59	7.66	6.97	7.54	7.65	7.63	7.28	7.58	8.14	6.62	0.10	0.04	-0.10	0.38*	-0.01	0.48*	-0.35
	Oppose	9.0%	9.1%	4.3%	9.5%	7.3%	8.3%	16.5%	8.2%	2.2%	5.9%	7.7%	7.4%	5.8%	21.2%	-0.8%	-6.9%	1.6%	-1.8%	0.1%	-2.4%	4.7%
	In the middle	11.5%	11.2%	5.3%	20.2%	9.2%	9.2%	15.7%	12.9%	21.0%	7.9%	14.1%	12.5%	9.7%	14.1%	1.3%	9.9%	2.6%	-6.1%	3.3%	0.5%	-1.7%
	Support	74.5%	79.8%	90.0%	65.8%	76.8%	75.3%	58.3%	76.8%	76.8%	86.3%	77.1%	78.0%	80.9%	59.0%	2.3%	-3.0%	-3.7%	11.2%	1.1%	5.6%	0.7%
	DK/NA	4.9%	0.0%	0.4%	4.4%	6.7%	7.2%	9.5%	2.2%	0.0%	0.0%	1.1%	2.2%	3.5%	5.7%	-2.8%	0.0%	-0.4%	-3.3%	-4.6%	-3.8%	-3.7%
Veterinarian assistants or allied veterinary professionals should be allowed to dispense antibiotics to farmers for a limited number of infections in livestock without a prescription.	Mean	6.99	5.67	7.26	7.25	7.64	7.28	6.63	7.25	7.72	7.29	7.57	7.39	7.67	5.58	0.25**	2.05***	0.03	0.32	-0.25	0.39	-1.05***
	Oppose	13.1%	31.0%	7.3%	8.5%	7.5%	9.6%	19.0%	10.8%	3.6%	7.9%	5.6%	11.1%	7.7%	30.3%	-2.3%	-27.3%	0.6%	-2.9%	3.6%	-1.9%	11.3%
	In the middle	12.0%	10.5%	8.8%	16.7%	8.9%	12.6%	15.0%	10.6%	14.7%	11.1%	11.1%	6.1%	7.7%	15.1%	-1.4%	4.2%	2.3%	-5.6%	-2.8%	-4.8%	0.1%
	Support	69.0%	58.5%	82.1%	70.3%	76.5%	65.4%	59.7%	75.6%	81.7%	81.0%	81.3%	78.4%	77.9%	51.0%	6.6%	23.1%	-1.1%	11.0%	1.9%	12.5%	-8.7%
	DK/NA	5.9%	0.0%	1.8%	4.6%	7.2%	12.4%	6.4%	3.1%	0.0%	0.0%	2.1%	4.5%	6.6%	3.6%	-2.8%	0.0%	-1.8%	-2.5%	-2.7%	-5.8%	-2.8%
Governments should develop monitoring (and evaluation) mechanisms to include informal sales of antibiotics.	Mean	8.05	7.58	7.98	8.02	8.07	8.49	7.98	8.51	8.68	8.44	8.09	8.93	9.16	7.26	0.45***	1.09***	0.46***	0.07	0.87***	0.67***	-0.73*
	Oppose	4.9%	7.7%	1.4%	4.7%	4.8%	4.2%	7.1%	4.2%	2.7%	1.1%	2.5%	2.4%	0.4%	18.7%	-0.6%	-5.0%	-0.3%	-2.2%	-2.4%	-3.8%	11.6%
	In the middle	8.6%	18.2%	6.4%	10.8%	6.6%	5.0%	6.7%	4.1%	2.3%	2.6%	8.8%	2.1%	0.6%	9.9%	-4.5%	-15.9%	-3.8%	-2.0%	-4.6%	-4.5%	3.1%
	Support	83.9%	72.1%	92.0%	82.0%	87.3%	89.3%	76.7%	90.6%	95.0%	96.3%	87.6%	95.0%	97.3%	68.4%	6.7%	22.8%	4.3%	5.5%	7.6%	8.0%	-8.4%
	DK/NA	2.6%	1.9%	0.3%	2.5%	1.3%	1.5%	9.5%	1.1%	0.0%	0.0%	1.1%	0.6%	1.7%	3.1%	-1.5%	-1.9%	-0.3%	-1.3%	-0.7%	0.3%	-6.4%
Governments should report the volumes of sales from these informal sources annually to the Global Antibiotic Surveillance System (a global monitoring system set up by the World Health Organization).	Mean	8.13	7.45	8.01	7.80	8.53	8.25	8.66	8.54	8.57	8.34	8.06	8.91	8.97	8.19	0.42***	1.13***	0.33*	0.25*	0.38**	0.72***	-0.47*
	Oppose	3.0%	5.3%	2.2%	4.6%	0.7%	4.1%	0.9%	1.5%	1.1%	0.3%	1.3%	0.8%	1.3%	4.8%	-1.4%	-4.2%	-1.9%	-3.2%	0.0%	-2.8%	3.9%
	In the middle	7.8%	15.7%	4.5%	12.4%	4.6%	7.2%	4.0%	5.2%	4.4%	3.7%	9.9%	2.3%	2.6%	9.9%	-2.7%	-11.3%	-0.8%	-2.5%	-2.3%	-4.6%	5.9%
	Support	84.0%	77.0%	93.4%	79.0%	91.4%	79.8%	83.3%	91.5%	94.5%	95.4%	87.1%	96.1%	91.7%	83.2%	7.5%	17.5%	2.1%	8.1%	4.7%	11.9%	0.0%
	DK/NA	5.2%	2.0%	0.0%	4.1%	3.3%	8.9%	11.8%	1.8%	0.0%	0.6%	1.7%	0.8%	4.4%	2.1%	-3.4%	-2.0%	0.6%	-2.4%	-2.4%	-4.5%	-9.8%
Governments should incentivise informal sellers to use only quality-assured antibiotics.	Mean	8.02	7.54	8.19	6.51	8.43	8.63	8.55	8.45	8.85	8.31	7.36	8.62	8.86	8.56	0.43***	1.31***	0.12	0.85***	0.20	0.23	0.01
	Oppose	7.8%	11.3%	4.1%	17.4%	2.5%	6.7%	6.3%	4.4%	2.8%	0.2%	8.1%	4.6%	4.5%	6.0%	-3.4%	-8.6%	-4.0%	-9.3%	2.1%	-2.2%	-0.3%
	In the middle	8.4%	16.1%	3.5%	20.6%	4.6%	2.3%	7.1%	5.4%	0.9%	1.4%	14.1%	6.3%	3.5%	6.5%	-3.0%	-15.3%	-2.1%	-6.5%	1.7%	1.2%	-0.5%
	Support	81.0%	72.5%	92.4%	59.0%	89.7%	85.3%	83.1%	88.9%	96.4%	98.1%	75.8%	89.0%	88.9%	85.7%	8.0%	23.8%	5.7%	16.8%	-0.7%	3.7%	2.6%
	DK/NA	2.8%	0.0%	0.0%	3.0%	3.3%	5.8%	3.5%	1.3%	0.0%	0.3%	2.0%	0.2%	3.1%	1.8%	-1.5%	0.0%	0.3%	-1.0%	-3.1%	-2.7%	-1.8%
An international initiative is needed to ensure affordable steady supply of quality-assured basic oral antibiotics in the low- and middle-income countries including local manufacturing.	Mean	8.12	7.52	7.81	7.87	8.41	8.58	8.32	8.58	8.57	8.30	8.09	8.85	9.02	8.49	0.47***	1.05***	0.49***	0.22	0.43***	0.44***	0.18
	Oppose	4.7%	17.5%	1.1%	3.4%	2.9%	2.7%	3.0%	1.6%	1.1%	0.8%	1.7%	1.4%	1.3%	3.6%	-3.1%	-16.4%	-0.3%	-1.7%	-1.5%	-1.4%	0.5%
	In the middle	6.8%	10.9%	3.3%	11.6%	5.3%	3.2%	8.8%	4.6%	2.8%	3.0%	9.2%	3.0%	2.5%	8.0%	-2.3%	-8.1%	-0.3%	-2.5%	-2.4%	-0.7%	-0.7%
	Support	84.9%	71.6%	94.9%	82.7%	89.5%	86.9%	81.1%	92.4%	96.0%	96.2%	88.0%	94.5%	91.7%	88.2%	7.5%	24.4%	1.3%	5.3%	5.0%	4.8%	7.1%
	DK/NA	3.5%	0.0%	0.6%	2.3%	2.4%	7.2%	7.1%	1.4%	0.0%	0.0%	1.1%	1.2%	4.4%	0.2%	-2.1%	0.0%	-0.6%	-1.2%	-1.2%	-2.8%	-6.9%
An international initiative is needed to ensure availability of proven/regulated point of care diagnostic tests to support appropriate antibiotic use in all countries.	Mean	8.21	8.14	7.89	7.77	8.32	8.57	8.46	8.65	8.73	8.39	8.11	8.90	8.91	8.81	0.45***	0.60***	0.50***	0.33**	0.58***	0.34**	0.35
	Oppose	3.1%	4.9%	0.8%	4.2%	3.7%	2.3%	2.9%	2.1%	0.9%	3.6%	1.4%	0.9%	3.3%	1.9%	-1.0%	-4.0%	2.8%	-2.8%	-2.7%	1.0%	-1.0%
	In the middle	5.6%	1.4%	7.7%	10.4%	2.3%	5.2%	7.1%	4.4%	2.0%	1.8%	8.8%	1.8%	3.5%	9.0%	-1.3%	0.6%	-5.9%	-1.6%	-0.4%	-1.7%	1.9%
	Support	88.1%	93.7%	90.9%	83.1%	91.3%	87.1%	82.5%	92.3%	96.6%	94.6%	88.7%	95.4%	90.5%	88.1%	4.2%	2.9%	3.7%	5.6%	4.1%	3.4%	5.6%
	DK/NA	3.2%	0.0%	0.6%	2.4%	2.8%	5.4%	7.4%	1.3%	0.5%	0.0%	1.1%	1.9%	2.7%	0.9%	-1.9%	0.5%	-0.6%	-1.2%	-0.9%	-2.7%	-6.6%
Governments should make currently available validated point of care, rapid diagnostic tests available at every primary health outlet.	Mean	8.48	7.84	8.22	8.23	8.48	8.86	9.15	8.71	8.43	8.62	8.29	8.77	9.00	9.08	0.23***	0.59***	0.39***	0.07	0.29*	0.14	-0.08
	Oppose	2.1%	6.7%	0.7%	3.0%	2.1%	0.5%	0.6%	1.7%	2.4%	0.4%	0.5%	3.5%	1.8%	1.2%	-0.4%	-4.3%	-0.2%	-2.5%	1.4%	1.3%	0.6%
	In the middle	6.9%	16.6%	4.3%	8.2%	8.0%	3.6%	2.6%	3.1%	2.2%	1.0%	8.4%	1.4%	1.7%	4.6%	-3.8%	-14.4%	-3.2%	0.3%	-6.6%	-2.0%	2.1%
	Support	88.9%	76.7%	95.1%	87.0%	87.9%	89.6%	96.0%	94.1%	95.4%	98.5%	89.6%	93.8%	94.0%	93.1%	5.2%	18.7%	3.4%	2.6%	5.8%	4.4%	-2.9%
	DK/NA	2.1%	0.0%	0.0%	1.8%	2.0%	6.2%	0.9%	1.2%	0.1%	0.0%	1.5%	1.4%	2.6%	1.1%	-0.9%	0.1%	0.0%	-0.4%	-0.6%	-3.7%	0.2%
Point of care rapid diagnostic tests need to be available and a part of care pathways, and should be reimbursed by the State or Medical Insurance Companies.	Mean	8.24	7.72	8.03	7.99	8.24	8.52	8.93	8.63	8.50	8.37	8.24	8.80	8.97	8.82	0.39***	0.79***	0.34**	0.25	0.56***	0.46**	-0.12
	Oppose	4.6%	16.1%	1.7%	4.4%	2.3%	2.2%	3.4%	1.4%	3.3%	0.6%	1.0%	2.8%	0.6%	0.6%	-3.2%	-12.8%	-1.0%	-3.4%	0.5%	-1.6%	-2.8%
	In the middle	5.4%	8.1%	2.4%	7.5%	5.4%	5.7%	3.5%	4.5%	1.2%	3.1%	9.5%	1.3%	3.9%	9.0%	-0.9%	-6.9%	0.8%	1.9%	-4.1%	-1.7%	5.5%
	Support	85.7%	75.8%	96.0%	85.6%	85.8%	83.1%	88.3%	91.6%	95.5%	96.3%	88.4%	94.5%	87.9%	87.7%	5.8%	19.7%	0.3%	2.8%	8.7%	4.9%	-0.6%
	DK/NA	4.2%	0.0%	0.0%	2.5%	6.5%	9.1%	4.8%	2.5%	0.0%	0.0%	1.1%	1.4%	7.6%	2.7%	-1.8%	0.0%	0.0%	-1.3%	-5.1%	-1.5%	-2.1%
There should be national certification standards from the manufacture of antibiotics in all countries for safe waste/outflow disposal, with penalties if standards are not met.	Mean	8.57	7.56	8.30	8.32	8.73	9.10	9.21	8.91	8.62	8.53	8.46	9.03	9.33	9.37	0.34***	1.06***	0.23	0.14	0.30*	0.23**	0.16
	Oppose	3.6%	17.1%	0.6%	3.2%	1.5%	0.4%	1.2%	0.8%	1.5%	0.0%	0.4%	2.3%	0.4%	0.2%	-2.7%	-15.6%	-0.6%	-2.8%	0.8%	0.0%	-1.0%
	In the middle	4.7%	7.8%	5.1%	7.0%	5.4%	2.7%	1.1%	3.0%	5.8%	0.6%	7.0%	2.5%	0.5%	3.0%	-1.7%	-2.0%	-4.5%	0.0%	-2.9%	-2.2%	1.9%
	Support	88.6%	73.9%	94.3%	87.9%	88.4%	91.1%	94.4%	95.3%	92.7%	99.4%	91.4%	94.1%	97.0%	96.3%	6.6%	18.9%	5.1%	3.5%	5.7%	5.9%	1.9%
	DK/NA	3.1%	1.3%	0.0%	1.8%	4.7%	5.8%	3.3%	0.9%	0.0%	0.0%	1.1%	1.1%	2.1%	0.4%	-2.2%	-1.3%	0.0%	-0.7%	-3.6%	-3.7%	-2.9%

*Note.* For results presented with asterisks (*), these asterisks indicate the results are statistically significant. The asterisks indicate the following: “*” indicates a p-value of less than 0.05, “**” indicates a p-value of less than 0.01, and “***” indicates a p-value of less than 0.001 resulting from a paired or independent t-test.

**Table 9.** Infection Prevention - mean ratings for each proposal, pre and post deliberation by country.

Proposal		Pre							Post							Difference						
	Measure	All	TZ	NG	ID	IN	CO	BR	All	TZ	NG	ID	IN	CO	BR	All	TZ	NG	ID	IN	CO	BR
National governments should prioritise infection prevention tools (clean water, safe sanitation and vaccination) to reduce the burden of infectious diseases.	Mean	8.88	7.94	8.31	8.65	8.96	9.54	9.58	9.12	8.58	8.83	8.67	9.26	9.59	9.58	0.24***	0.64***	0.53***	0.02	0.30**	0.05	0.01
	Oppose	3.9%	19.3%	2.0%	2.2%	1.6%	0.4%	0.6%	0.3%	1.4%	0.2%	0.4%	0.0%	0.0%	0.2%	-3.5%	-17.9%	-1.9%	-1.7%	-1.6%	-0.4%	-0.4%
	In the middle	3.0%	2.5%	1.6%	6.2%	3.1%	1.8%	3.1%	2.8%	3.5%	1.0%	6.8%	2.0%	0.0%	4.6%	-0.2%	1.0%	-0.6%	0.6%	-1.1%	-1.8%	1.5%
	Support	92.2%	76.1%	96.3%	90.2%	94.5%	96.6%	95.9%	96.4%	95.1%	98.8%	91.6%	98.0%	98.6%	94.7%	4.2%	19.0%	2.5%	1.4%	3.5%	1.9%	-1.1%
	DK/NA	1.0%	2.2%	0.0%	1.4%	0.8%	1.2%	0.4%	0.6%	0.0%	0.0%	1.1%	0.0%	1.4%	0.4%	-0.5%	-2.2%	0.0%	-0.3%	-0.8%	0.2%	0.0%
Everyone should have access to clean water and safe sanitation (WASH) in their community and health system.	Mean	9.08	8.09	8.52	8.75	9.24	9.79	9.75	9.27	8.84	8.95	8.57	9.48	9.79	9.73	0.19***	0.75***	0.43***	-0.18	0.24*	0.01	-0.02
	Oppose	3.5%	17.2%	3.5%	2.2%	0.9%	0.0%	0.0%	0.3%	0.0%	0.0%	0.9%	0.9%	0.0%	0.0%	-3.2%	-17.2%	-3.5%	-1.3%	0.1%	0.0%	0.0%
	In the middle	2.0%	1.6%	3.3%	4.8%	1.0%	0.7%	1.0%	1.7%	1.6%	0.0%	7.1%	2.0%	0.0%	0.3%	-0.2%	0.1%	-3.3%	2.3%	0.9%	-0.7%	-0.7%
	Support	93.7%	79.9%	92.2%	91.6%	98.1%	98.2%	98.6%	97.0%	98.4%	100.0%	90.2%	97.1%	96.9%	99.7%	3.3%	18.5%	7.8%	-1.4%	-1.0%	-1.3%	1.1%
	DK/NA	0.9%	1.3%	1.0%	1.4%	0.0%	1.1%	0.4%	0.9%	0.0%	0.0%	1.8%	0.0%	3.1%	0.0%	0.1%	-1.3%	-1.0%	0.3%	0.0%	2.0%	-0.4%
Specific government funding should go to healthcare facilities to ensure that all have access to clean water for hand hygiene.	Mean	8.90	7.93	8.59	8.60	9.06	9.49	9.46	9.06	8.56	8.86	8.56	9.22	9.54	9.39	0.16***	0.63**	0.27*	-0.04	0.15	0.05	-0.07
	Oppose	3.1%	16.6%	0.0%	2.7%	1.4%	0.0%	0.0%	1.1%	4.1%	0.8%	0.4%	1.9%	0.0%	0.0%	-1.9%	-12.5%	0.8%	-2.3%	0.5%	0.0%	0.0%
	In the middle	2.7%	3.7%	1.3%	5.8%	4.1%	1.3%	0.4%	3.6%	4.3%	0.6%	6.4%	2.8%	2.4%	6.1%	0.9%	0.6%	-0.7%	0.6%	-1.3%	1.1%	5.7%
	Support	93.5%	79.2%	98.7%	90.1%	94.0%	97.6%	99.1%	94.6%	91.5%	98.6%	91.7%	95.2%	95.8%	93.6%	1.0%	12.3%	-0.1%	1.7%	1.2%	-1.7%	-5.5%
	DK/NA	0.7%	0.5%	0.0%	1.4%	0.5%	1.1%	0.4%	0.7%	0.1%	0.0%	1.4%	0.1%	1.8%	0.2%	0.0%	-0.4%	0.0%	0.0%	-0.4%	0.6%	-0.2%
Immunization for WHO-recommended childhood vaccination should be scaled up towards universal/full coverage, to capture all children.	Mean	8.74	7.46	8.37	8.48	8.89	9.50	9.48	9.08	9.00	8.82	8.45	9.16	9.43	9.56	0.34***	1.54***	0.45***	-0.03	0.27*	-0.07	0.08
	Oppose	2.7%	11.3%	2.4%	2.1%	0.7%	0.8%	0.9%	1.1%	1.6%	0.0%	1.2%	1.1%	1.8%	0.5%	-1.7%	-9.6%	-2.4%	-0.9%	0.4%	1.0%	-0.3%
	In the middle	6.3%	23.6%	0.7%	8.0%	6.0%	1.1%	1.7%	1.4%	0.3%	1.3%	6.5%	0.0%	0.4%	0.6%	-4.9%	-23.3%	0.6%	-1.5%	-6.0%	-0.7%	-1.1%
	Support	88.4%	64.6%	96.0%	88.0%	91.4%	91.9%	95.1%	96.5%	97.9%	98.7%	91.2%	98.5%	94.5%	98.4%	8.0%	33.3%	2.8%	3.2%	7.1%	2.7%	3.3%
	DK/NA	2.5%	0.5%	1.0%	1.9%	1.8%	6.2%	2.4%	1.0%	0.1%	0.0%	1.1%	0.4%	3.2%	0.4%	-1.5%	-0.4%	-1.0%	-0.7%	-1.5%	-2.9%	-1.9%
An adult programme of vaccination should be created by national governments to ensure vaccination of vulnerable populations for influenza and pneumonia.	Mean	8.52	7.55	7.98	8.04	8.81	9.20	9.22	8.84	8.45	8.57	8.30	8.98	9.16	9.46	0.33***	0.90***	0.60***	0.26*	0.17	-0.04	0.24
	Oppose	2.9%	6.0%	1.8%	3.9%	2.2%	1.8%	2.1%	1.2%	1.3%	0.0%	0.9%	0.1%	3.9%	0.2%	-1.6%	-4.7%	-1.8%	-3.0%	-2.1%	2.1%	-1.9%
	In the middle	5.5%	7.8%	1.5%	10.5%	5.4%	4.2%	4.1%	3.7%	8.3%	0.0%	7.2%	2.9%	2.1%	2.6%	-1.8%	0.5%	-1.5%	-3.3%	-2.5%	-2.1%	-1.5%
	Support	89.6%	84.7%	94.2%	83.1%	90.8%	90.8%	93.2%	93.6%	90.4%	99.4%	90.2%	95.0%	90.9%	96.3%	4.0%	5.7%	5.2%	7.1%	4.2%	0.1%	3.1%
	DK/NA	2.0%	1.4%	2.5%	2.5%	1.6%	3.1%	0.7%	1.5%	0.0%	0.6%	1.8%	2.0%	3.0%	0.9%	-0.5%	-1.4%	-1.9%	-0.7%	0.4%	-0.1%	0.2%
Influenza vaccination should be incentivized for patient-facing healthcare workers.	Mean	8.49	7.69	7.76	8.22	8.61	8.98	9.49	8.76	8.33	8.29	8.21	8.87	9.14	9.57	0.27***	0.64**	0.53***	-0.02	0.26*	0.16	0.08
	Oppose	3.2%	10.0%	1.2%	2.1%	1.5%	4.1%	0.6%	2.1%	1.5%	0.0%	3.0%	3.3%	3.8%	0.0%	-1.1%	-8.5%	-1.2%	0.9%	1.9%	-0.3%	-0.6%
	In the middle	5.6%	11.0%	6.0%	10.1%	3.5%	3.2%	1.3%	5.0%	6.8%	6.6%	7.6%	2.9%	3.8%	3.5%	-0.6%	-4.2%	0.6%	-2.5%	-0.7%	0.6%	2.2%
	Support	88.9%	78.2%	92.3%	84.6%	88.2%	91.8%	96.4%	90.8%	91.7%	93.4%	88.3%	86.1%	90.1%	96.5%	1.9%	13.5%	1.1%	3.8%	-2.2%	-1.7%	0.1%
	DK/NA	2.4%	0.8%	0.5%	3.3%	6.8%	0.9%	1.6%	2.1%	0.0%	0.0%	1.1%	7.7%	2.3%	0.0%	-0.3%	-0.8%	-0.5%	-2.1%	1.0%	1.4%	-1.6%
Hospital accreditation should be linked to infection prevention, such as using hand washing rates as a factor.	Mean	8.59	8.17	8.11	8.39	8.69	9.11	8.89	8.76	8.54	8.54	8.25	9.05	9.02	9.07	0.17***	0.37*	0.43***	-0.14	0.35**	-0.09	0.18
	Oppose	2.9%	6.1%	2.1%	2.2%	2.7%	2.3%	2.1%	1.2%	1.0%	0.0%	2.7%	0.2%	1.8%	1.2%	-1.7%	-5.1%	-2.1%	0.5%	-2.5%	-0.5%	-0.9%
	In the middle	4.3%	4.2%	2.7%	6.9%	3.5%	3.3%	5.8%	4.1%	8.0%	1.6%	6.9%	0.5%	3.7%	4.9%	-0.2%	3.8%	-1.1%	0.0%	-3.0%	0.3%	-0.9%
	Support	90.0%	89.6%	94.2%	88.8%	89.7%	91.2%	86.0%	92.6%	91.0%	98.4%	88.8%	95.9%	91.3%	89.6%	2.5%	1.4%	4.2%	-0.1%	6.2%	0.1%	3.6%
	DK/NA	2.8%	0.0%	1.0%	2.1%	4.1%	3.2%	6.1%	2.2%	0.0%	0.0%	1.6%	3.4%	3.2%	4.2%	-0.6%	0.0%	-1.0%	-0.5%	-0.6%	0.1%	-1.9%
Hospitals should be mandated to publish their hand hygiene rates annually.	Mean	8.19	7.71	7.92	8.50	8.69	7.86	8.47	8.43	8.13	8.53	8.45	8.88	7.87	8.73	0.23***	0.43*	0.62***	-0.05	0.19	0.02	0.26
	Oppose	5.9%	17.8%	1.4%	1.2%	2.4%	9.0%	4.1%	1.9%	0.6%	0.3%	0.4%	1.0%	4.9%	3.3%	-4.0%	-17.1%	-1.0%	-0.8%	-1.5%	-4.1%	-0.8%
	In the middle	8.8%	10.4%	7.7%	6.6%	5.3%	12.9%	9.2%	7.5%	9.3%	0.6%	5.3%	2.7%	14.7%	10.7%	-1.3%	-1.2%	-7.0%	-1.2%	-2.6%	1.8%	1.5%
	Support	81.1%	71.8%	90.7%	90.8%	88.9%	67.6%	79.7%	87.9%	90.1%	96.1%	93.1%	95.8%	72.4%	84.4%	6.8%	18.3%	5.4%	2.3%	6.9%	4.8%	4.7%
	DK/NA	4.2%	0.0%	0.3%	1.4%	3.5%	10.5%	7.0%	2.7%	0.0%	2.9%	1.1%	0.6%	8.0%	1.6%	-1.5%	0.0%	2.6%	-0.3%	-2.9%	-2.5%	-5.4%
Farms should ensure good infection prevention and biosecurity	Mean	8.55	7.72	8.11	8.33	8.73	9.01	9.17	8.87	8.26	8.53	8.34	9.05	9.24	9.60	0.32***	0.54*	0.42***	0.00	0.32**	0.23*	0.43***
	Oppose	3.4%	18.0%	1.0%	0.8%	1.1%	1.2%	0.6%	0.7%	2.1%	0.0%	0.3%	1.6%	0.4%	0.0%	-2.7%	-15.9%	-1.0%	-0.5%	0.5%	-0.8%	-0.6%
	In the middle	4.6%	7.8%	3.5%	7.6%	2.9%	4.5%	1.4%	3.8%	8.9%	3.8%	8.0%	1.0%	2.2%	0.4%	-0.7%	1.1%	0.3%	0.3%	-1.9%	-2.3%	-1.0%
	Support	89.9%	73.4%	95.5%	87.5%	92.8%	92.1%	96.0%	94.2%	88.5%	96.2%	90.0%	96.0%	95.6%	97.8%	4.3%	15.1%	0.7%	2.5%	3.2%	3.5%	1.9%
	DK/NA	2.1%	0.7%	0.0%	4.1%	3.2%	2.2%	2.0%	1.2%	0.5%	0.0%	1.8%	1.4%	1.8%	1.7%	-0.9%	-0.2%	0.0%	-2.4%	-1.8%	-0.4%	-0.3%
Farming, including fish, should use vaccination	Mean	7.98	7.62	7.80	8.19	8.23	7.65	8.52	8.11	8.21	7.87	8.08	8.21	7.88	8.53	0.13	0.59**	0.08	-0.11	-0.02	0.23	0.01
	Oppose	6.0%	12.4%	1.2%	1.2%	6.8%	9.6%	3.7%	5.1%	7.4%	3.0%	2.4%	7.3%	7.1%	2.0%	-1.0%	-5.0%	1.8%	1.1%	0.5%	-2.5%	-1.7%
	In the middle	6.3%	7.7%	4.2%	8.1%	6.0%	6.2%	6.1%	7.7%	5.5%	3.7%	8.3%	5.6%	13.3%	8.2%	1.4%	-2.2%	-0.5%	0.2%	-0.4%	7.1%	2.1%
	Support	77.5%	79.8%	93.2%	84.4%	79.6%	61.7%	71.8%	80.5%	87.1%	90.1%	87.1%	82.2%	65.0%	77.3%	3.0%	7.4%	-3.1%	2.7%	2.5%	3.3%	5.5%
	DK/NA	10.2%	0.1%	1.4%	6.3%	7.6%	22.5%	18.4%	6.7%	0.0%	3.2%	2.3%	4.9%	14.6%	12.4%	-3.4%	-0.1%	1.8%	-4.0%	-2.7%	-7.8%	-6.0%
Farm workers should be taught hand hygiene	Mean	8.80	7.98	8.35	8.71	8.89	9.21	9.49	9.03	8.40	8.86	8.68	9.02	9.33	9.76	0.23***	0.42*	0.51***	-0.03	0.13	0.12	0.27**
	Oppose	4.2%	19.0%	0.0%	1.2%	3.7%	3.0%	0.0%	1.2%	2.0%	0.4%	0.4%	1.4%	2.3%	0.0%	-3.1%	-17.1%	0.4%	-0.8%	-2.3%	-0.7%	0.0%
	In the middle	3.1%	4.0%	2.6%	4.8%	2.9%	3.0%	1.1%	3.9%	8.7%	0.9%	5.4%	5.5%	2.1%	1.3%	0.8%	4.7%	-1.7%	0.6%	2.6%	-0.9%	0.2%
	Support	91.4%	76.8%	97.4%	91.0%	91.9%	92.9%	96.7%	94.0%	89.3%	97.6%	93.0%	91.7%	94.5%	98.0%	2.7%	12.5%	0.2%	2.1%	-0.1%	1.6%	1.3%
	DK/NA	1.3%	0.2%	0.0%	3.0%	1.6%	1.1%	2.2%	1.0%	0.1%	1.0%	1.1%	1.4%	1.2%	0.7%	-0.4%	-0.2%	1.0%	-1.9%	-0.2%	0.1%	-1.4%

*Note.* For results presented with asterisks (*), these asterisks indicate the results are statistically significant. The asterisks indicate the following: “*” indicates a p-value of less than 0.05, “**” indicates a p-value of less than 0.01, and “***” indicates a p-value of less than 0.001 resulting from a paired or independent t-test.

**Table 10.** Food Security - mean ratings for each proposal, pre and post deliberation by country.

Proposal		Pre							Post							Difference						
	Measure	All	TZ	NG	ID	IN	CO	BR	All	TZ	NG	ID	IN	CO	BR	All	TZ	NG	ID	IN	CO	BR
An identifying label should accompany food that is produced without the use of routine antibiotics	Mean	8.17	8.02	7.73	7.39	8.19	8.83	8.67	8.58	8.50	8.26	7.87	8.42	9.19	9.08	0.41***	0.48*	0.54***	0.48***	0.23	0.36**	0.42*
	Oppose	4.6%	6.1%	4.0%	5.7%	5.3%	3.0%	3.9%	2.9%	2.5%	1.0%	3.1%	5.9%	1.3%	3.7%	-1.7%	-3.7%	-2.9%	-2.6%	0.6%	-1.7%	-0.1%
	In the middle	8.1%	10.0%	3.0%	16.5%	7.4%	4.6%	8.8%	4.8%	3.0%	6.8%	11.4%	3.9%	1.9%	2.8%	-3.3%	-7.0%	3.8%	-5.1%	-3.6%	-2.7%	-6.1%
	Support	80.7%	77.6%	92.0%	71.2%	79.0%	82.9%	80.8%	90.3%	94.5%	92.1%	83.5%	85.5%	93.6%	92.3%	9.5%	16.9%	0.1%	12.3%	6.5%	10.8%	11.5%
	DK/NA	6.6%	6.2%	1.0%	6.6%	8.2%	9.6%	6.5%	2.0%	0.0%	0.0%	2.0%	4.7%	3.2%	1.2%	-4.6%	-6.2%	-1.0%	-4.6%	-3.5%	-6.4%	-5.3%
Large-scale food suppliers – supermarkets, restaurant chains, intensive farming etc., – should be incentivized to promote the use of food produced without routine antibiotics.	Mean	7.88	7.76	7.56	6.95	8.19	8.44	8.37	8.24	8.56	8.16	7.26	8.14	8.74	8.60	0.36***	0.80***	0.60***	0.32	-0.05	0.31	0.22
	Oppose	5.8%	6.8%	4.9%	9.2%	6.5%	3.9%	4.2%	4.1%	3.1%	0.8%	5.3%	8.7%	3.3%	3.2%	-1.7%	-3.7%	-4.2%	-3.9%	2.1%	-0.6%	-1.0%
	In the middle	8.6%	9.3%	6.5%	16.6%	4.9%	6.7%	8.7%	7.7%	1.9%	6.3%	15.2%	6.0%	8.5%	7.9%	-0.8%	-7.4%	-0.2%	-1.5%	1.2%	1.8%	-0.9%
	Support	77.8%	83.7%	87.9%	68.0%	81.9%	71.8%	74.9%	84.1%	94.5%	91.0%	77.9%	82.7%	80.2%	80.4%	6.3%	10.8%	3.0%	9.9%	0.8%	8.3%	5.5%
	DK/NA	7.9%	0.2%	0.6%	6.1%	6.6%	17.5%	12.2%	4.1%	0.5%	1.9%	1.6%	2.6%	8.0%	8.6%	-3.8%	0.3%	1.3%	-4.5%	-4.0%	-9.6%	-3.7%
Large-scale food suppliers – supermarkets, restaurant chains, intensive farming etc., – should be regulated to promote the use of food produced without routine antibiotics.	Mean	7.81	7.46	7.69	7.76	7.95	8.37	7.48	8.11	8.44	7.97	7.89	8.16	8.61	7.35	0.29***	0.98***	0.29	0.13	0.21	0.24	-0.13
	Oppose	7.4%	10.9%	5.1%	2.9%	9.7%	5.0%	12.3%	4.8%	1.4%	0.6%	1.2%	9.0%	3.8%	12.5%	-2.7%	-9.5%	-4.5%	-1.7%	-0.8%	-1.2%	0.3%
	In the middle	10.0%	12.1%	6.6%	13.6%	7.1%	8.8%	13.1%	8.7%	9.1%	6.7%	12.0%	3.5%	7.9%	14.6%	-1.3%	-3.0%	0.1%	-1.6%	-3.5%	-0.9%	1.6%
	Support	75.3%	75.3%	87.7%	79.0%	76.5%	70.7%	63.5%	82.0%	89.5%	92.7%	85.3%	83.0%	79.6%	61.8%	6.6%	14.2%	5.0%	6.3%	6.5%	8.9%	-1.8%
	DK/NA	7.3%	1.8%	0.6%	4.5%	6.7%	15.6%	11.2%	4.6%	0.0%	0.0%	1.5%	4.5%	8.7%	11.1%	-2.7%	-1.8%	-0.6%	-3.0%	-2.2%	-6.9%	-0.1%
Governments should regulate farmers to establish infection prevention programs on farms (including vaccination) linked to an inspection program.	Mean	8.38	7.37	7.99	8.09	8.63	8.95	9.08	8.66	8.49	8.42	8.13	8.59	9.11	9.14	0.28***	1.12***	0.43***	0.04	-0.03	0.17	0.06
	Oppose	2.8%	9.7%	1.7%	1.2%	0.6%	2.6%	1.7%	1.1%	1.3%	0.6%	0.5%	2.6%	0.4%	1.2%	-1.7%	-8.4%	-1.1%	-0.8%	2.0%	-2.2%	-0.5%
	In the middle	7.6%	22.6%	6.0%	8.6%	4.1%	4.1%	3.5%	3.7%	4.0%	1.5%	7.4%	2.4%	3.2%	4.5%	-3.9%	-18.6%	-4.5%	-1.2%	-1.7%	-0.9%	1.0%
	Support	86.7%	66.2%	92.3%	87.2%	93.0%	90.1%	87.2%	93.3%	94.6%	97.8%	90.6%	93.5%	92.9%	90.1%	6.6%	28.4%	5.6%	3.4%	0.5%	2.9%	2.9%
	DK/NA	2.9%	1.5%	0.0%	2.9%	2.3%	3.2%	7.6%	1.9%	0.1%	0.0%	1.5%	1.5%	3.5%	4.2%	-1.0%	-1.4%	0.0%	-1.4%	-0.8%	0.2%	-3.4%
Group prevention therapy with antibiotics for sick animals should be under the management of a veterinarian or allied professional.	Mean	8.45	7.47	7.81	8.21	8.45	9.09	9.46	8.72	8.30	8.01	8.24	8.82	9.31	9.43	0.27***	0.84***	0.20	0.04	0.38***	0.23*	-0.03
	Oppose	2.5%	9.7%	1.1%	0.8%	4.1%	0.3%	0.0%	1.5%	3.2%	2.9%	0.8%	0.4%	0.9%	1.4%	-1.0%	-6.5%	1.8%	0.0%	-3.7%	0.6%	1.4%
	In the middle	7.3%	20.1%	8.6%	9.9%	3.6%	3.2%	1.3%	3.9%	3.8%	4.4%	8.2%	3.9%	2.3%	1.5%	-3.3%	-16.3%	-4.2%	-1.6%	0.2%	-0.9%	0.3%
	Support	87.0%	69.2%	88.3%	86.4%	88.7%	90.5%	96.1%	92.9%	93.0%	91.8%	89.8%	91.9%	94.2%	96.6%	5.9%	23.9%	3.5%	3.4%	3.2%	3.7%	0.5%
	DK/NA	3.2%	1.1%	2.0%	2.9%	3.5%	6.0%	2.6%	1.6%	0.0%	1.0%	1.1%	3.8%	2.6%	0.4%	-1.6%	-1.1%	-1.0%	-1.8%	0.3%	-3.4%	-2.2%
Governments should ban from food production systems the use of antibiotics that are critically important for humans.	Mean	7.33	6.67	7.31	7.32	7.95	8.01	6.28	7.82	8.49	7.37	7.92	7.84	8.40	6.46	0.49***	1.82***	0.06	0.60***	-0.11	0.39	0.19
	Oppose	10.2%	13.8%	9.2%	6.2%	6.4%	8.2%	19.7%	7.9%	2.6%	7.5%	1.0%	8.9%	8.0%	19.2%	-2.4%	-11.1%	-1.7%	-5.2%	2.5%	-0.3%	-0.6%
	In the middle	12.3%	19.2%	4.6%	17.8%	9.5%	7.8%	17.8%	9.9%	6.1%	11.3%	14.9%	4.5%	6.7%	17.8%	-2.4%	-13.1%	6.7%	-3.0%	-4.9%	-1.1%	-0.1%
	Support	69.3%	67.1%	84.7%	66.4%	74.8%	71.3%	48.6%	76.6%	91.3%	80.0%	83.0%	79.0%	78.3%	46.8%	7.3%	24.2%	-4.7%	16.6%	4.3%	7.0%	-1.8%
	DK/NA	8.2%	0.0%	1.6%	9.6%	9.3%	12.6%	13.9%	5.6%	0.0%	1.3%	1.1%	7.5%	7.0%	16.3%	-2.5%	0.0%	-0.3%	-8.4%	-1.8%	-5.6%	2.4%

*Note.* For results presented with asterisks (*), these asterisks indicate the results are statistically significant. The asterisks indicate the following: “*” indicates a p-value of less than 0.05, “**” indicates a p-value of less than 0.01, and “***” indicates a p-value of less than 0.001 resulting from a paired or independent t-test.

**Table 11.** Awareness and Education - mean ratings for each proposal, pre and post deliberation by country.

Proposal		Pre							Post							Difference						
	Measure	All	TZ	NG	ID	IN	CO	BR	All	TZ	NG	ID	IN	CO	BR	All	TZ	NG	ID	IN	CO	BR
Food produced with the routine use of antibiotics should be identified by an international standard, such as a hazard-warning symbol or label.	Mean	8.46	8.26	7.82	8.32	8.45	9.09	8.70	8.70	8.32	8.56	8.42	8.63	9.15	9.04	0.24***	0.05	0.74***	0.10	0.19	0.06	0.33
	Oppose	2.7%	3.1%	2.6%	0.8%	3.2%	3.3%	3.0%	1.8%	3.4%	1.6%	1.3%	2.6%	0.4%	1.8%	-0.9%	0.3%	-1.0%	0.5%	-0.6%	-2.9%	-1.2%
	In the middle	5.8%	8.6%	3.2%	9.2%	4.8%	5.5%	4.0%	4.2%	5.2%	0.9%	6.8%	5.3%	3.5%	4.1%	-1.5%	-3.4%	-2.3%	-2.4%	0.5%	-2.0%	0.1%
	Support	88.4%	86.1%	94.2%	88.1%	88.0%	87.1%	86.9%	91.1%	91.4%	97.1%	90.8%	89.3%	88.9%	90.4%	2.8%	5.3%	2.9%	2.7%	1.3%	1.8%	3.6%
	DK/NA	3.2%	2.2%	0.0%	1.9%	4.0%	4.1%	6.2%	2.9%	0.0%	0.4%	1.1%	2.8%	7.2%	3.7%	-0.3%	-2.2%	0.4%	-0.8%	-1.3%	3.1%	-2.5%
Hand hygiene and infection prevention should be part of the standard curriculum in schools from the start of schooling through to graduation.	Mean	8.72	8.21	8.37	8.29	8.82	9.12	9.35	8.95	8.45	8.52	8.32	9.31	9.46	9.43	0.23***	0.24	0.14	0.03	0.49***	0.34***	0.08
	Oppose	2.2%	8.0%	0.3%	1.7%	0.8%	1.2%	2.3%	0.6%	0.5%	0.6%	1.1%	0.4%	0.3%	0.7%	-1.6%	-7.5%	0.2%	-0.6%	-0.4%	-1.0%	-1.5%
	In the middle	5.4%	8.3%	2.9%	8.4%	6.6%	3.8%	2.8%	3.1%	4.0%	2.7%	7.6%	0.3%	1.7%	3.5%	-2.3%	-4.2%	-0.2%	-0.8%	-6.3%	-2.1%	0.7%
	Support	91.1%	83.7%	96.7%	88.1%	89.8%	93.5%	93.4%	95.8%	95.5%	96.7%	90.2%	99.0%	96.9%	95.5%	4.7%	11.7%	-0.1%	2.1%	9.2%	3.4%	2.1%
	DK/NA	1.3%	0.0%	0.0%	1.8%	2.8%	1.5%	1.6%	0.5%	0.0%	0.0%	1.1%	0.3%	1.2%	0.3%	-0.8%	0.0%	0.0%	-0.7%	-2.5%	-0.3%	-1.2%
Awareness campaigns should focus on the risks and harms of taking unnecessary antibiotics on humans, food security, and the environment.	Mean	8.50	7.97	8.08	7.82	8.70	9.05	9.20	8.82	8.18	8.59	8.13	9.13	9.36	9.31	0.32***	0.21	0.51***	0.32*	0.43***	0.31***	0.11
	Oppose	2.7%	6.1%	3.5%	3.0%	1.4%	1.6%	1.6%	1.5%	6.8%	0.0%	1.1%	0.0%	0.3%	1.7%	-1.3%	0.7%	-3.5%	-1.9%	-1.4%	-1.3%	0.1%
	In the middle	8.2%	18.7%	1.7%	16.3%	5.6%	3.6%	6.1%	3.2%	5.1%	2.5%	7.7%	2.1%	1.1%	1.9%	-5.0%	-13.7%	0.8%	-8.7%	-3.6%	-2.5%	-4.2%
	Support	86.8%	75.2%	94.8%	76.5%	91.4%	90.5%	89.2%	93.9%	88.1%	96.5%	89.7%	97.9%	95.4%	94.2%	7.1%	12.9%	1.7%	13.2%	6.4%	4.8%	5.0%
	DK/NA	2.3%	0.0%	0.0%	4.2%	1.5%	4.3%	3.1%	1.4%	0.0%	1.0%	1.5%	0.1%	3.3%	2.2%	-0.9%	0.0%	1.0%	-2.6%	-1.4%	-1.0%	-0.9%
National governments should increase funding for awareness campaigns into vaccine hesitancy and improving understanding about people’s behaviours relating to vaccination.	Mean	8.42	7.89	8.05	7.84	8.62	8.95	8.95	8.80	8.41	8.53	7.97	9.11	9.19	9.44	0.39***	0.53***	0.48***	0.14	0.48***	0.24*	0.49***
	Oppose	2.3%	5.4%	2.0%	3.3%	1.3%	1.5%	1.0%	0.8%	1.8%	0.4%	2.0%	0.4%	0.3%	0.0%	-1.5%	-3.6%	-1.6%	-1.3%	-0.9%	-1.2%	-1.0%
	In the middle	6.8%	7.8%	5.4%	10.8%	5.3%	5.9%	6.4%	3.0%	4.6%	1.4%	9.1%	0.9%	1.6%	1.2%	-3.9%	-3.2%	-4.0%	-1.8%	-4.4%	-4.3%	-5.2%
	Support	88.3%	86.8%	92.6%	82.3%	89.6%	88.4%	90.0%	95.2%	93.6%	98.2%	87.1%	98.6%	95.2%	97.6%	6.8%	6.9%	5.6%	4.9%	9.0%	6.8%	7.6%
	DK/NA	2.5%	0.0%	0.0%	3.6%	3.8%	4.2%	2.6%	1.1%	0.0%	0.0%	1.8%	0.1%	2.9%	1.2%	-1.4%	0.0%	0.0%	-1.9%	-3.6%	-1.3%	-1.4%

*Note.* For results presented with asterisks (*), these asterisks indicate the results are statistically significant. The asterisks indicate the following: “*” indicates a p-value of less than 0.05, “**” indicates a p-value of less than 0.01, and “***” indicates a p-value of less than 0.001 resulting from a paired or independent t-test.

**Table 12.** Targets - mean ratings for each proposal, pre and post deliberation by country.

Proposal	Pre								Post							Difference						
	Measure	All	TZ	NG	ID	IN	CO	BR	All	TZ	NG	ID	IN	CO	BR	All	TZ	NG	ID	IN	CO	BR
There should be global targets to address bacterial resistance to antibiotics, just like global targets for climate.	Mean	8.30	7.77	7.80	7.94	8.40	8.87	8.90	8.67	8.50	8.30	8.19	8.86	9.16	8.88	0.37***	0.73***	0.50***	0.25**	0.47***	0.29*	-0.02
	Oppose	3.7%	17.3%	2.9%	0.8%	1.3%	1.5%	0.9%	1.7%	1.1%	0.6%	0.9%	1.1%	1.7%	4.9%	-2.0%	-16.1%	-2.2%	0.1%	-0.2%	0.2%	4.0%
	In the middle	6.9%	5.8%	4.9%	11.0%	9.0%	5.5%	5.0%	4.4%	5.0%	3.1%	8.6%	3.2%	3.0%	4.4%	-2.5%	-0.8%	-1.8%	-2.5%	-5.8%	-2.5%	-0.7%
	Support	85.1%	75.3%	91.9%	83.7%	87.3%	83.1%	89.1%	92.3%	93.9%	96.3%	89.4%	93.9%	90.4%	90.2%	7.2%	18.6%	4.3%	5.8%	6.6%	7.3%	1.1%
	DK/NA	4.3%	1.7%	0.3%	4.5%	2.4%	9.9%	5.0%	1.6%	0.0%	0.0%	1.1%	1.8%	4.9%	0.5%	-2.6%	-1.7%	-0.3%	-3.4%	-0.5%	-5.0%	-4.4%
There should be a global target to reduce human deaths from antibiotic resistance.	Mean	8.47	7.87	7.99	8.18	8.52	9.13	8.92	8.76	8.34	8.51	8.19	8.95	9.32	9.03	0.29***	0.48*	0.51***	0.02	0.43***	0.19*	0.11
	Oppose	2.8%	8.2%	4.0%	2.0%	0.8%	2.5%	0.2%	0.8%	2.8%	0.4%	1.0%	0.6%	0.0%	0.9%	-2.0%	-5.5%	-3.5%	-1.0%	-0.2%	-2.5%	0.8%
	In the middle	8.1%	21.0%	3.3%	7.7%	9.1%	3.0%	7.4%	3.5%	3.1%	1.5%	6.9%	3.4%	2.7%	4.0%	-4.6%	-17.9%	-1.8%	-0.8%	-5.7%	-0.3%	-3.4%
	Support	85.2%	70.8%	92.8%	85.7%	86.7%	88.0%	84.7%	94.3%	93.3%	98.1%	90.4%	94.7%	94.6%	94.3%	9.1%	22.5%	5.3%	4.7%	8.0%	6.6%	9.7%
	DK/NA	3.9%	0.0%	0.0%	4.6%	3.5%	6.4%	7.8%	1.3%	0.9%	0.0%	1.8%	1.4%	2.7%	0.7%	-2.5%	0.9%	0.0%	-2.9%	-2.1%	-3.8%	-7.1%
There should be a global target to stop the use of medically critical antibiotics in the food chain.	Mean	7.86	7.28	7.37	7.93	8.25	8.56	7.46	8.36	8.54	8.06	8.07	8.89	8.89	7.31	0.51***	1.26***	0.69***	0.14	0.64***	0.33*	-0.15
	Oppose	5.3%	3.8%	8.2%	1.8%	3.3%	4.4%	11.2%	4.1%	3.7%	1.5%	2.0%	2.1%	3.3%	12.9%	-1.2%	-0.1%	-6.6%	0.2%	-1.3%	-1.1%	1.7%
	In the middle	12.5%	29.4%	7.8%	12.5%	9.1%	8.3%	11.8%	7.4%	4.1%	7.3%	9.7%	1.8%	6.2%	17.1%	-5.1%	-25.3%	-0.6%	-2.8%	-7.4%	-2.1%	5.3%
	Support	74.8%	65.2%	82.4%	79.8%	85.8%	72.7%	60.3%	84.5%	92.2%	91.2%	86.8%	93.2%	83.0%	59.2%	9.7%	27.0%	8.8%	7.0%	7.4%	10.3%	-1.1%
	DK/NA	7.4%	1.6%	1.6%	5.9%	1.8%	14.7%	16.8%	4.0%	0.0%	0.0%	1.5%	3.0%	7.5%	10.8%	-3.4%	-1.6%	-1.6%	-4.4%	1.2%	-7.1%	-6.0%
There should be a global target to stop the use of antibiotics for growth promotion and infection prevention without veterinary advice	Mean	7.96	7.80	7.37	7.58	8.26	8.25	8.43	8.33	8.21	7.90	7.99	8.55	8.71	8.54	0.37***	0.41*	0.53**	0.41**	0.29	0.46**	0.11
	Oppose	6.0%	3.3%	7.3%	7.2%	4.6%	7.8%	5.2%	3.1%	1.2%	4.9%	2.3%	2.2%	4.1%	3.7%	-2.9%	-2.1%	-2.5%	-4.9%	-2.5%	-3.7%	-1.4%
	In the middle	8.7%	12.2%	9.0%	12.0%	5.5%	7.2%	7.6%	7.6%	8.6%	5.2%	12.8%	6.1%	4.2%	10.8%	-1.1%	-3.6%	-3.8%	0.7%	0.6%	-3.0%	3.2%
	Support	80.5%	83.2%	83.6%	75.8%	83.1%	76.9%	81.3%	85.6%	90.1%	87.1%	83.8%	88.9%	83.6%	80.2%	5.1%	7.0%	3.4%	8.0%	5.8%	6.7%	-1.1%
	DK/NA	4.8%	1.3%	0.0%	5.0%	6.8%	8.1%	5.9%	3.6%	0.0%	2.9%	1.1%	2.8%	8.1%	5.2%	-1.1%	-1.3%	2.9%	-3.8%	-3.9%	0.0%	-0.7%
Antibiotic resistance should be included as a cause of death on all national death certificates to enable collection of data on mortality.	Mean	7.91	7.01	7.41	7.74	8.32	8.67	8.06	8.51	8.52	8.02	7.98	8.63	9.14	8.62	0.60***	1.52***	0.61***	0.24	0.31*	0.47***	0.56*
	Oppose	4.9%	11.4%	3.7%	1.5%	4.2%	2.8%	7.7%	2.8%	3.3%	1.9%	1.9%	2.4%	1.4%	6.9%	-2.1%	-8.0%	-1.8%	0.5%	-1.8%	-1.4%	-0.8%
	In the middle	8.8%	15.1%	11.0%	13.9%	3.4%	4.7%	7.8%	5.4%	4.6%	7.1%	10.8%	4.5%	3.6%	2.8%	-3.4%	-10.6%	-3.9%	-3.1%	1.1%	-1.1%	-5.0%
	Support	80.8%	71.1%	83.7%	80.2%	86.8%	83.3%	76.4%	88.9%	92.1%	91.0%	85.6%	89.8%	88.2%	86.8%	8.1%	21.1%	7.3%	5.4%	3.1%	4.9%	10.3%
	DK/NA	5.5%	2.4%	1.7%	4.4%	5.7%	9.2%	8.1%	2.9%	0.0%	0.0%	1.7%	3.3%	6.8%	3.6%	-2.7%	-2.4%	-1.7%	-2.7%	-2.4%	-2.4%	-4.5%

*Note.* For results presented with asterisks (*), these asterisks indicate the results are statistically significant. The asterisks indicate the following: “*” indicates a p-value of less than 0.05, “**” indicates a p-value of less than 0.01, and “***” indicates a p-value of less than 0.001 resulting from a paired or independent t-test.

**Table 13.** Surveillance - mean ratings for each proposal, pre and post deliberation by country.

Proposal	Pre								Post							Difference						
	Measure	All	TZ	NG	ID	IN	CO	BR	All	TZ	NG	ID	IN	CO	BR	All	TZ	NG	ID	IN	CO	BR
National surveillance programs should be in place to monitor - infection rates, bacterial types, antibiotic use, and antibiotic resistance – in humans, animals (including fish) and water systems.	Mean	8.42	7.95	7.98	8.20	8.54	8.94	8.77	8.82	8.69	8.46	8.18	8.95	9.25	9.30	0.40***	0.73***	0.48***	-0.02	0.41***	0.31**	0.53***
	Oppose	2.2%	5.5%	3.9%	0.3%	2.1%	0.4%	1.9%	0.8%	1.7%	0.6%	1.0%	0.5%	0.3%	1.0%	-1.4%	-3.7%	-3.4%	0.6%	-1.6%	-0.1%	-0.9%
	In the middle	6.9%	18.6%	2.6%	10.1%	3.2%	4.2%	5.2%	3.0%	2.7%	0.2%	8.5%	1.1%	2.4%	3.9%	-3.9%	-16.0%	-2.5%	-1.6%	-2.0%	-1.8%	-1.3%
	Support	87.8%	73.7%	93.4%	87.8%	93.2%	86.7%	90.5%	94.4%	95.6%	99.3%	88.8%	96.9%	95.2%	89.5%	6.6%	21.8%	5.8%	1.1%	3.7%	8.5%	-1.0%
	DK/NA	3.1%	2.2%	0.0%	1.8%	1.6%	8.8%	2.4%	1.8%	0.0%	0.0%	1.8%	1.5%	2.2%	5.5%	-1.3%	-2.2%	0.0%	-0.1%	-0.1%	-6.6%	3.1%
International funding support should be available for surveillance in countries willing to invest in laboratory capacity, monitoring, and reporting and where systems are not yet available.	Mean	8.36	7.58	8.01	8.14	8.44	8.99	8.80	8.77	8.42	8.65	7.98	8.85	9.31	9.31	0.42***	0.85***	0.65***	-0.16	0.41***	0.31***	0.50***
	Oppose	3.5%	16.6%	1.0%	1.6%	2.5%	0.8%	0.6%	1.2%	2.0%	0.0%	2.7%	0.9%	1.2%	0.2%	-2.3%	-14.6%	-1.0%	1.1%	-1.6%	0.4%	-0.4%
	In the middle	6.4%	8.9%	2.3%	9.7%	7.6%	3.5%	7.8%	3.8%	6.4%	1.3%	10.2%	2.4%	1.1%	2.8%	-2.6%	-2.5%	-1.0%	0.5%	-5.2%	-2.4%	-5.0%
	Support	86.9%	74.4%	96.6%	86.5%	87.6%	90.1%	83.3%	93.8%	91.6%	97.7%	86.0%	96.5%	95.5%	94.3%	6.9%	17.1%	1.1%	-0.5%	8.9%	5.5%	11.0%
	DK/NA	3.2%	0.1%	0.0%	2.2%	2.3%	5.6%	8.3%	1.2%	0.0%	1.0%	1.1%	0.2%	2.2%	2.7%	-2.0%	-0.1%	1.0%	-1.1%	-2.2%	-3.5%	-5.6%
Surveillance data should be reported nationally, regionally, and to the World Health Organization, Food and Agriculture Organisation, and the World Organisation for Animal Health.	Mean	8.31	7.74	7.79	8.05	8.41	8.83	8.92	8.72	8.37	8.27	8.23	8.92	9.15	9.27	0.41***	0.63***	0.48***	0.18	0.51***	0.32**	0.35*
	Oppose	1.9%	2.5%	3.1%	2.5%	2.9%	0.6%	0.0%	1.0%	1.9%	0.0%	0.4%	1.1%	1.8%	0.2%	-0.9%	-0.6%	-3.1%	-2.1%	-1.8%	1.2%	0.2%
	In the middle	8.6%	23.9%	6.1%	9.8%	4.8%	5.2%	4.7%	3.0%	5.0%	2.6%	7.5%	1.3%	1.3%	1.6%	-5.5%	-18.9%	-3.5%	-2.3%	-3.5%	-3.9%	-3.1%
	Support	86.5%	73.6%	90.9%	85.5%	87.8%	91.0%	86.9%	94.3%	93.1%	96.5%	90.9%	97.0%	93.0%	95.3%	7.8%	19.5%	5.6%	5.4%	9.1%	2.0%	8.4%
	DK/NA	3.1%	0.0%	0.0%	2.2%	4.5%	3.3%	8.4%	1.7%	0.0%	1.0%	1.1%	0.7%	3.9%	2.9%	-1.4%	0.0%	1.0%	-1.0%	-3.8%	0.6%	-5.5%
Laboratory systems for measuring antibiotic resistance in bacteria for humans and animals should be integrated with shared One Health resources.	Mean	8.31	7.90	7.78	8.19	8.41	8.85	8.75	8.59	8.74	8.30	8.19	8.60	9.00	8.72	0.28***	0.84***	0.52***	0.00	0.19	0.15	-0.03
	Oppose	1.9%	5.8%	1.2%	1.6%	2.4%	0.6%	0.8%	1.5%	4.2%	0.9%	1.7%	0.7%	1.2%	1.0%	-0.4%	-1.5%	-0.4%	0.1%	-1.7%	0.5%	0.2%
	In the middle	5.8%	6.7%	4.3%	8.6%	6.0%	5.3%	3.8%	4.3%	3.0%	1.5%	6.5%	4.2%	4.1%	6.9%	-1.4%	-3.7%	-2.8%	-2.1%	-1.8%	-1.3%	3.2%
	Support	85.4%	87.3%	94.0%	87.6%	87.9%	73.1%	87.1%	89.8%	91.4%	97.7%	90.1%	92.1%	82.3%	87.6%	4.4%	4.1%	3.7%	2.5%	4.2%	9.2%	0.5%
	DK/NA	6.9%	0.3%	0.5%	2.2%	3.7%	21.0%	8.4%	4.3%	1.4%	0.0%	1.7%	2.9%	12.5%	4.5%	-2.5%	1.1%	-0.5%	-0.5%	-0.8%	-8.5%	-3.8%
Data from point of care rapid diagnostic tests should be incorporated into routine national surveillance reporting.	Mean	8.26	7.77	7.70	8.11	8.40	8.60	8.88	8.65	8.65	8.33	8.24	8.62	9.00	9.00	0.39***	0.88***	0.63***	0.13	0.23	0.39***	0.12
	Oppose	2.3%	5.0%	2.2%	2.0%	2.8%	2.2%	0.0%	1.4%	2.0%	1.5%	0.9%	2.0%	1.5%	0.4%	-0.9%	-3.1%	-0.7%	-1.1%	-0.9%	-0.7%	0.4%
	In the middle	6.0%	8.7%	2.9%	9.1%	4.1%	5.6%	6.7%	4.4%	4.9%	2.7%	7.9%	2.3%	3.4%	6.3%	-1.6%	-3.8%	-0.3%	-1.2%	-1.7%	-2.2%	-0.4%
	Support	86.8%	85.8%	92.6%	86.7%	85.9%	82.4%	89.5%	92.3%	92.5%	95.8%	90.1%	92.8%	91.9%	90.4%	5.4%	6.8%	3.2%	3.4%	6.9%	9.6%	0.8%
	DK/NA	4.8%	0.5%	2.3%	2.3%	7.2%	9.8%	3.8%	1.9%	0.6%	0.0%	1.1%	2.9%	3.2%	2.9%	-2.9%	0.1%	-2.3%	-1.1%	-4.3%	-6.7%	-0.9%

*Note.* For results presented with asterisks (*), these asterisks indicate the results are statistically significant. The asterisks indicate the following: “*” indicates a p-value of less than 0.05, “**” indicates a p-value of less than 0.01, and “***” indicates a p-value of less than 0.001 resulting from a paired or independent t-test.

We did see variation in certain types of proposals by country/region. Overall, Tanzanian participants experienced the greatest shifts in opinion through the deliberation process. For example, their level of support for 13/14 equity, access, and stewardship proposals increased dramatically by 0.596 to 2.049 points ( Table 8). The two largest shifts in opinion among Tanzanians for access-related proposals were in support for registered pharmacists prescribing (41.3% to 72.5% support) and veterinarians (58.5% to 81.7% support) to be allowed to dispense antibiotics without a prescription.

The reverse trend, i.e., increasing opposition for certain access proposals was evident in Brazilian participants. Before deliberation, they were less supportive of pharmacists, veterinarian assistants, and informal antibiotic sellers being able to dispense antibiotics without a prescription compared to the other 5 countries, an opposition to which increased after deliberation, (support decreasing by 0.72-1.05 points, Table 8). They expressed concern about a lack of ongoing medical supervision with informal antibiotic distribution, captured by one Brazilian participant who stated “ *That’s why I’m in favour of using antibiotics as such, always under medical supervision, not by a health professional, not by an informal salesperson or just by taking them at home without any real knowledge. I believe that this medical supervision is necessary even if you are hospitalized yourself, you were prescribed it by a doctor and you need to go back to him to tell him that you are experiencing other symptoms.*”

**Figure 3.**
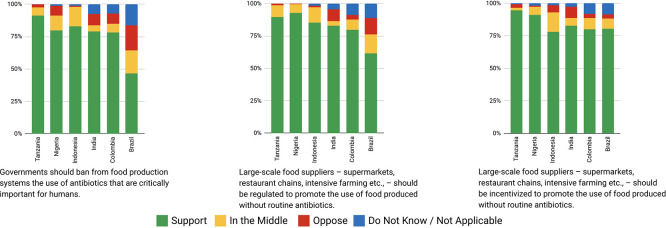
Levels of support for proposals relating to food security after deliberation expressed as percentages for each country.

Brazil, and to a lesser extent Colombia were also less supportive of proposals relating to the use of antibiotics in food production than African and Asian countries. While food security proposals received general collective support, the most being for governments to regulate farmers to establish infection prevention programmes on farms (93.3%), the proposal for governments to ban use of antibiotics that are critically important for humans from food production systems, saw only 46.8% (6.464 points) of Brazilian participants supporting the proposal after deliberation, in contrast to the other end of the spectrum, where Tanzanian participants registered 94.6% support ( Figure 3). The proposal for farmers to use vaccination, which received 80.5% collective support, was least supported by Colombian (65% support) and Brazilian participants (77.3%) after deliberation ( Table 9). Vaccination itself seemed not to be the issue, as both countries registered >90% support for scale-up immunization for WHO-recommended childhood vaccination to full coverage. Brazilian participants were also not supportive of large-scale food suppliers being regulated to promote the use of food produced without routine (animal growth promotion and large-scale prevention) antibiotics relative to other countries but were, however, supportive of incentivising such food suppliers to promote food produced without routine antibiotics and of antibiotic therapy being managed by veterinary professionals (96.6% support, Table 10). One Brazilian participant’s view was illustrative, suggesting a preference for management and oversight rather than outright bans: “ *I think there needs to be supervision, right?...because sometimes antibiotics are used when animals don’t need them. I think that when there is a need, they should be used and there should be supervision*” and “ *I’m also in favour of the use of antibiotics in animals, right? As long as there’s this control, then I think that if everything is monitored properly, I’m in favour.*”

## Knowledge gains relating to antibiotic resistance and political efficacy

**Figure 4.**
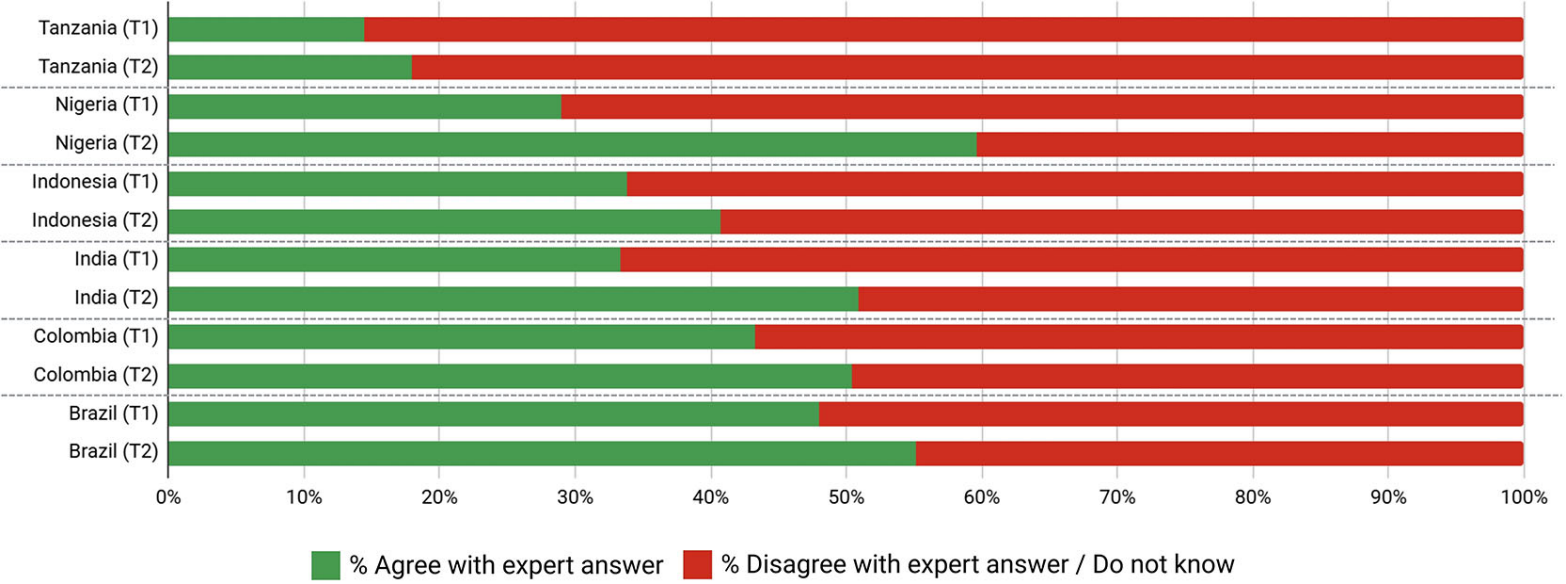
Knowledge scores for 6 questions relating to antibiotic resistance before and after deliberation, by country. Results are expressed as percentages.

**Figure 5.**
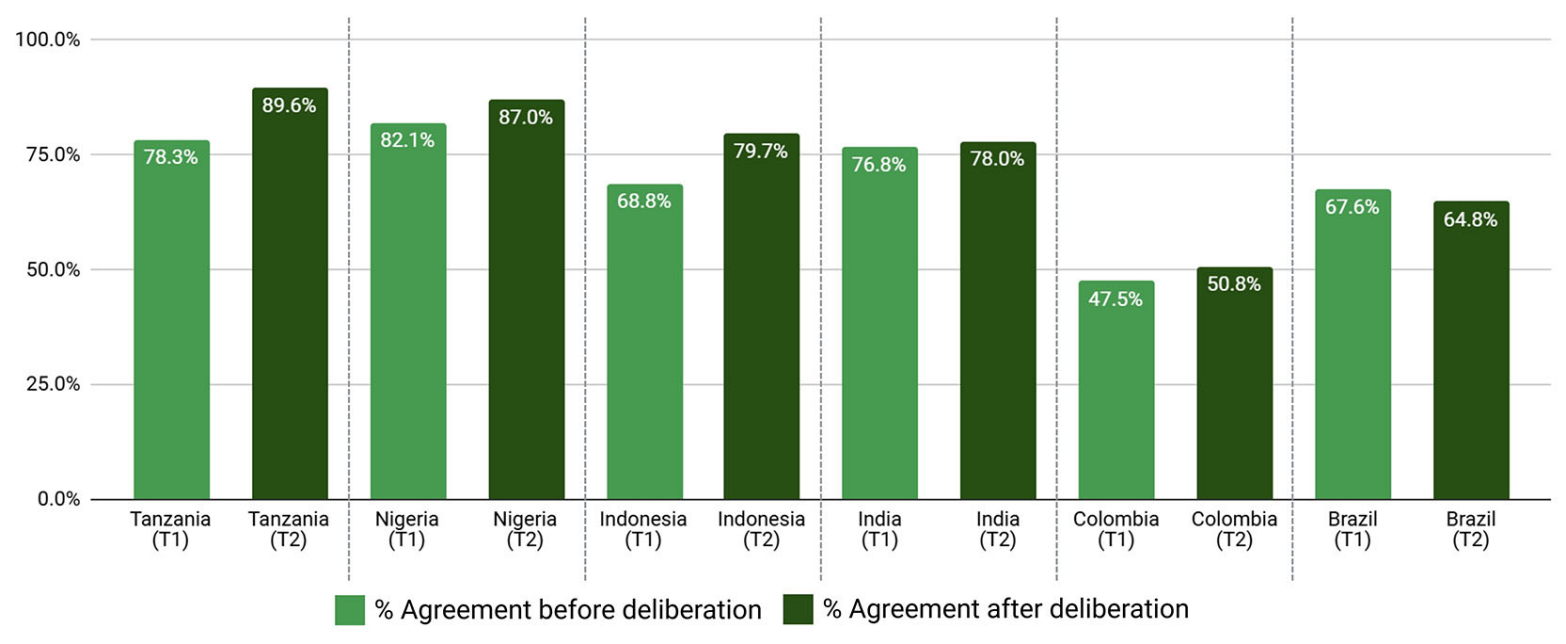
Percentage agreement with statements relating to internal political efficacy before and after deliberation.

Increases in agreement with the expert advisory group’s consensus answers for knowledge questions ( Table 14) indicates that participants gained knowledge about antibiotic resistance through deliberation ( Figure 4). Nigeria, India, Colombia, and Indonesia increased their percentage of correct responses for all 6 questions, and Brazilian and Tanzanian participants for five each. Political efficacy i.e., a person’s trust in their ability to change the government and belief that they can understand and influence political affairs also increased after deliberation across countries ( Figure 5).

**Table 14.** Knowledge Questions.

Question	Expert Answer
What does antibiotic resistance mean? Choose only one answer	a. Medicine that is working against your body to make you more sick b. A virus causing infection in your body is able to resist the action of an antibiotic c. Antibiotic manufacturers have become resistant to producing antibiotics d. Bacteria causing an infection are able to resist the action of an antibiotic * e. Don’t know
When was the latest discovery of a new antibiotic class that has reached the market?	a. 2019 b. 2005 c. 1999 d. 1987 * e. 1956 f. Don’t Know
Which of the following statements are TRUE about antibiotics? Choose only one answer.	a. Antibiotics can kill bacteria. b. Antibiotics have side-effects such as diarrhoea, nausea and vomiting. c. Antibiotics cannot kill viruses. d. People can be allergic to antibiotics. e. All of the above * f. Don’t know
Which of the following is a way to prevent infection, in order to reduce antibiotic resistance? Choose only one answer	a. One of the largest bugs, in size, on earth that causes the worse colds b. A bug with ‘super’ abilities to eat large amount of bacteria to cure illnesses c. The bacteria that can be killed by any antibiotic d. The most resistant types of bacteria almost always found in hospitals * e. Don’t know
Which of the following statements is a fact about antibiotic resistance	a. a. More children under the age of 5 years die from lack of access to an antibiotic than die of antibiotic resistant bacterial infection b. Antibiotic use has been associated with obesity later in life c. Antibiotic use has been associated with developing inflammatory bowel disease (ulcerative colitis and Crohn’s Disease) in later life d. Antibiotic misuse in humans is a major cause of increasing antibiotic resistance e. All of the above * f. Don’t Know

* Denotes the correct answer to each question as ascertained by the Expert Advisory Panel.

### Impact of deliberation

By examining differences between intervention and control group changes before and after deliberation, the impact of deliberation on participants’ attitudes towards proposals can be inferred ( Table 15). Given that so many proposals were highly rated before deliberation, there might have been ceiling effects that prevented them from going significantly higher, thus limiting the impact of the deliberation on changes in proposal ratings. For example, participants that viewed proposals positively before the deliberation may have had their views confirmed through deliberation and did not significantly change their survey ratings of proposals.

**Table 15.** Differences between the intervention group changes and the control group changes before and after deliberation for each of the 45 proposals for each country. The p-value column indicates the significance of the analysis for each row, comparison of the treatment and control group for the particular question.

Country	Proposal	Control n	Participant n	Control Mean	Participant Mean	Statistic	p-value	Adjusted p
Brazil	p01	178	188	9.23	9.462	-1.482	0.139	0.287
Brazil	p02	178	188	8.869	8.955	-0.447	0.655	0.808
Brazil	p03	178	188	7.162	6.884	0.793	0.428	0.612
Brazil	p04	178	188	6.158	6.544	-1.042	0.298	0.473
Brazil	p05	178	188	7.207	7.052	0.49	0.624	0.788
Brazil	p06	178	188	6.407	6.693	-0.793	0.429	0.612
Brazil	p07	178	188	7.919	7.815	0.369	0.712	0.851
Brazil	p08	178	188	8.469	8.744	-1.328	0.185	0.34
Brazil	p09	178	188	8.337	8.742	-1.588	0.113	0.247
Brazil	p10	178	188	8.343	8.486	-0.625	0.532	0.711
Brazil	p11	178	188	8.328	8.569	-1.05	0.295	0.471
Brazil	p12	178	188	9.318	9.188	0.898	0.37	0.558
Brazil	p13	178	188	8.949	9.028	-0.468	0.64	0.798
Brazil	p14	178	188	9.264	9.311	-0.349	0.727	0.858
Brazil	p15	178	188	9.568	9.647	-0.726	0.469	0.639
Brazil	p16	178	188	9.808	9.786	0.301	0.763	0.882
Brazil	p17	178	188	9.631	9.604	0.235	0.814	0.916
Brazil	p18	178	188	9.384	9.524	-0.899	0.369	0.558
Brazil	p19	178	188	9.386	9.333	0.365	0.715	0.851
Brazil	p20	178	188	9.394	9.5	-0.765	0.445	0.629
Brazil	p21	178	188	9.186	9.112	0.434	0.664	0.815
Brazil	p22	178	188	8.865	8.852	0.059	0.953	0.984
Brazil	p23	178	188	9.491	9.387	0.777	0.438	0.622
Brazil	p24	178	188	8.563	8.584	-0.084	0.933	0.977
Brazil	p25	178	188	9.52	9.538	-0.148	0.882	0.97
Brazil	p26	178	188	9.042	8.966	0.38	0.704	0.845
Brazil	p27	178	188	8.481	8.454	0.102	0.919	0.977
Brazil	p28	178	188	7.974	7.525	1.425	0.155	0.304
Brazil	p29	178	188	9.198	9.193	0.028	0.978	0.992
Brazil	p30	178	188	9.351	9.486	-1.037	0.301	0.475
Brazil	p31	178	188	7.247	6.477	2.023	0.044	0.116
Brazil	p32	178	188	9.036	8.988	0.263	0.792	0.906
Brazil	p33	178	188	9.44	9.44	-0.002	0.999	1
Brazil	p34	178	188	9.224	9.3	-0.466	0.641	0.798
Brazil	p35	178	188	9.08	9.033	0.242	0.809	0.914
Brazil	p36	178	188	9.219	9.074	0.874	0.383	0.571
Brazil	p37	178	188	9.285	9.08	1.187	0.236	0.403
Brazil	p38	178	188	7.975	7.742	0.736	0.462	0.636
Brazil	p39	178	188	8.795	8.682	0.501	0.617	0.782
Brazil	p40	178	188	8.524	8.415	0.417	0.677	0.827
Brazil	p41	178	188	9.099	8.978	0.745	0.457	0.636
Brazil	p42	178	188	8.964	8.948	0.094	0.926	0.977
Brazil	p43	178	188	9.121	9.073	0.301	0.763	0.882
Brazil	p44	178	188	9.073	8.905	0.972	0.332	0.512
Brazil	p45	178	188	9.198	9.116	0.535	0.593	0.766
Colombia	p01	185	275	8.797	8.996	-1.143	0.254	0.426
Colombia	p02	185	275	8.916	8.715	1.201	0.231	0.399
Colombia	p03	185	275	7.415	7.516	-0.387	0.699	0.843
Colombia	p04	185	275	6.983	6.996	-0.044	0.965	0.987
Colombia	p05	185	275	7.809	7.682	0.524	0.601	0.769
Colombia	p06	185	275	7.186	7.181	0.019	0.985	0.993
Colombia	p07	185	275	8.797	8.478	1.826	0.069	0.166
Colombia	p08	185	275	8.347	8.265	0.405	0.686	0.83
Colombia	p09	185	275	8.807	8.582	1.127	0.261	0.432
Colombia	p10	185	275	8.712	8.606	0.612	0.541	0.719
Colombia	p11	185	275	8.709	8.521	1.087	0.278	0.455
Colombia	p12	185	275	9.078	8.885	1.429	0.154	0.303
Colombia	p13	185	275	8.594	8.652	-0.334	0.738	0.867
Colombia	p14	185	275	8.966	9.065	-0.669	0.504	0.683
Colombia	p15	185	275	9.486	9.52	-0.296	0.767	0.882
Colombia	p16	185	275	9.723	9.787	-1.001	0.317	0.495
Colombia	p17	185	275	9.568	9.581	-0.145	0.885	0.97
Colombia	p18	185	275	9.525	9.525	0	1	1
Colombia	p19	185	275	9.238	9.23	0.053	0.958	0.984
Colombia	p20	185	275	9.17	9.151	0.116	0.908	0.977
Colombia	p21	185	275	9.211	9.199	0.101	0.919	0.977
Colombia	p22	185	275	8.452	7.908	2.343	0.02	0.059
Colombia	p23	185	275	9.05	9.063	-0.094	0.925	0.977
Colombia	p24	185	275	7.814	7.742	0.246	0.806	0.914
Colombia	p25	185	275	9.244	9.311	-0.47	0.639	0.798
Colombia	p26	185	275	9.135	8.755	2.417	0.016	0.051
Colombia	p27	185	275	8.679	8.457	1.059	0.29	0.471
Colombia	p28	185	275	8.415	8.403	0.059	0.953	0.984
Colombia	p29	185	275	9.12	8.865	1.706	0.089	0.207
Colombia	p30	185	275	8.989	9.099	-0.796	0.426	0.612
Colombia	p31	185	275	7.904	7.884	0.076	0.939	0.979
Colombia	p32	185	275	9.235	9.011	1.665	0.097	0.217
Colombia	p33	185	275	9.374	9.158	1.68	0.094	0.212
Colombia	p34	185	275	9.201	9.098	0.81	0.418	0.607
Colombia	p35	185	275	8.983	8.996	-0.084	0.933	0.977
Colombia	p36	185	275	9.006	8.881	0.747	0.456	0.636
Colombia	p37	185	275	8.943	9.085	-0.903	0.367	0.558
Colombia	p38	185	275	8.47	8.533	-0.299	0.765	0.882
Colombia	p39	185	275	8.462	8.44	0.098	0.922	0.977
Colombia	p40	185	275	8.729	8.6	0.666	0.506	0.683
Colombia	p41	185	275	9.029	8.984	0.299	0.765	0.882
Colombia	p42	185	275	9	9.077	-0.555	0.579	0.759
Colombia	p43	185	275	8.988	8.903	0.542	0.588	0.766
Colombia	p44	185	275	8.794	8.819	-0.14	0.889	0.97
Colombia	p45	185	275	8.653	8.66	-0.038	0.97	0.988
India	p01	160	231	8.558	8.827	-1.492	0.137	0.284
India	p02	160	231	8.189	8.726	-2.823	0.005	0.021
India	p03	160	231	7.955	7.715	1.002	0.317	0.495
India	p04	160	231	7.297	7.441	-0.528	0.598	0.769
India	p05	160	231	7.316	7.61	-1.156	0.249	0.422
India	p06	160	231	7.182	7.53	-1.377	0.169	0.317
India	p07	160	231	8.057	8.173	-0.555	0.579	0.759
India	p08	160	231	8.195	8.453	-1.448	0.149	0.298
India	p09	160	231	8.19	8.538	-1.685	0.093	0.212
India	p10	160	231	8.323	8.48	-0.859	0.391	0.577
India	p11	160	231	8.449	8.371	0.458	0.647	0.802
India	p12	160	231	8.5	8.533	-0.203	0.84	0.929
India	p13	160	231	8.447	8.324	0.733	0.464	0.636
India	p14	160	231	8.516	8.698	-1.055	0.292	0.471
India	p15	160	231	8.885	9.017	-0.827	0.409	0.597
India	p16	160	231	9	9.229	-1.448	0.149	0.298
India	p17	160	231	8.747	8.991	-1.392	0.165	0.315
India	p18	160	231	8.633	8.886	-1.446	0.149	0.298
India	p19	160	231	8.327	8.779	-2.465	0.014	0.046
India	p20	160	231	8.338	8.598	-1.374	0.171	0.317
India	p21	160	231	8.365	8.641	-1.63	0.104	0.229
India	p22	160	231	8.51	8.644	-0.737	0.462	0.636
India	p23	160	231	8.484	8.76	-1.688	0.092	0.212
India	p24	160	231	7.762	8.128	-1.535	0.126	0.265
India	p25	160	231	8.646	8.819	-0.986	0.325	0.504
India	p26	160	231	7.953	8.215	-1.145	0.253	0.426
India	p27	160	231	7.799	8.102	-1.282	0.201	0.352
India	p28	160	231	7.716	7.898	-0.739	0.46	0.636
India	p29	160	231	8.245	8.542	-1.729	0.085	0.201
India	p30	160	231	8.431	8.5	-0.409	0.683	0.83
India	p31	160	231	7.912	7.849	0.254	0.8	0.911
India	p32	160	231	8.461	8.518	-0.348	0.728	0.858
India	p33	160	231	8.777	8.796	-0.12	0.905	0.977
India	p34	160	231	8.545	8.789	-1.414	0.158	0.308
India	p35	160	231	8.358	8.655	-1.635	0.103	0.229
India	p36	160	231	8.539	8.531	0.052	0.959	0.984
India	p37	160	231	8.455	8.575	-0.633	0.527	0.709
India	p38	160	231	8.123	8.354	-1.103	0.271	0.446
India	p39	160	231	8.006	8.293	-1.287	0.199	0.352
India	p40	160	231	7.832	8.361	-2.537	0.012	0.041
India	p41	160	231	8.293	8.507	-1.19	0.235	0.403
India	p42	160	231	8.201	8.482	-1.546	0.123	0.261
India	p43	160	231	8.25	8.456	-1.128	0.26	0.432
India	p44	160	231	8.196	8.302	-0.579	0.563	0.745
India	p45	160	231	8.238	8.335	-0.515	0.607	0.773
Indonesia	p01	200	198	8.53	8.204	2.222	0.027	0.076
Indonesia	p02	200	198	8.388	8.021	2.311	0.021	0.062
Indonesia	p03	200	198	8.219	7.661	3.061	0.002	0.011
Indonesia	p04	200	198	7.909	7.13	3.719	0	0.002
Indonesia	p05	200	198	7.855	6.853	4.872	0	0
Indonesia	p06	200	198	8.057	7.222	4.31	0	0
Indonesia	p07	200	198	8.236	7.969	1.503	0.134	0.28
Indonesia	p08	200	198	8.25	7.791	2.514	0.012	0.042
Indonesia	p09	200	198	7.69	6.547	4.97	0	0
Indonesia	p10	200	198	8.327	7.912	2.575	0.01	0.039
Indonesia	p11	200	198	8.411	7.809	3.663	0	0.002
Indonesia	p12	200	198	8.497	8.231	1.709	0.088	0.207
Indonesia	p13	200	198	8.382	8	2.37	0.018	0.056
Indonesia	p14	200	198	8.452	8.308	0.878	0.38	0.571
Indonesia	p15	200	198	8.894	8.602	1.935	0.054	0.139
Indonesia	p16	200	198	8.726	8.73	-0.024	0.981	0.992
Indonesia	p17	200	198	8.754	8.566	1.278	0.202	0.352
Indonesia	p18	200	198	8.709	8.497	1.376	0.17	0.317
Indonesia	p19	200	198	8.601	8.062	3.325	0.001	0.005
Indonesia	p20	200	198	8.585	8.223	2.313	0.021	0.062
Indonesia	p21	200	198	8.575	8.39	1.284	0.2	0.352
Indonesia	p22	200	198	8.665	8.485	1.28	0.201	0.352
Indonesia	p23	200	198	8.566	8.346	1.566	0.118	0.256
Indonesia	p24	200	198	8.602	8.219	2.569	0.011	0.039
Indonesia	p25	200	198	8.621	8.699	-0.539	0.59	0.766
Indonesia	p26	200	198	8.032	7.438	3.149	0.002	0.008
Indonesia	p27	200	198	7.689	6.973	3.475	0.001	0.004
Indonesia	p28	200	198	8.061	7.742	1.851	0.065	0.161
Indonesia	p29	200	198	8.402	8.114	1.993	0.047	0.123
Indonesia	p30	200	198	8.425	8.234	1.333	0.183	0.339
Indonesia	p31	200	198	8.062	7.387	3.458	0.001	0.004
Indonesia	p32	200	198	8.551	8.338	1.46	0.145	0.297
Indonesia	p33	200	198	8.422	8.292	0.864	0.388	0.576
Indonesia	p34	200	198	8.308	7.826	2.656	0.008	0.032
Indonesia	p35	200	198	8.317	7.885	2.737	0.007	0.026
Indonesia	p36	200	198	8.396	7.995	2.675	0.008	0.031
Indonesia	p37	200	198	8.416	8.216	1.317	0.189	0.344
Indonesia	p38	200	198	8.077	7.942	0.837	0.403	0.592
Indonesia	p39	200	198	8.137	7.676	2.595	0.01	0.037
Indonesia	p40	200	198	8.169	7.811	2.278	0.023	0.066
Indonesia	p41	200	198	8.422	8.215	1.395	0.164	0.315
Indonesia	p42	200	198	8.355	8.16	1.289	0.198	0.352
Indonesia	p43	200	198	8.34	8.092	1.552	0.121	0.26
Indonesia	p44	200	198	8.372	8.216	1.05	0.294	0.471
Indonesia	p45	200	198	8.32	8.124	1.288	0.199	0.352
Nigeria	p01	210	203	7.608	8.158	-3.078	0.002	0.01
Nigeria	p02	210	203	7.364	8.005	-3.618	0	0.002
Nigeria	p03	210	203	7.268	7.97	-3.53	0	0.003
Nigeria	p04	210	203	6.895	7.724	-4.041	0	0.001
Nigeria	p05	210	203	7.25	7.851	-3.194	0.002	0.007
Nigeria	p06	210	203	6.781	7.411	-2.962	0.003	0.014
Nigeria	p07	210	203	7.558	7.98	-2.371	0.018	0.056
Nigeria	p08	210	203	7.584	7.901	-1.871	0.062	0.155
Nigeria	p09	210	203	7.795	8.227	-2.536	0.012	0.041
Nigeria	p10	210	203	7.55	7.881	-1.973	0.049	0.128
Nigeria	p11	210	203	7.373	7.975	-3.349	0.001	0.005
Nigeria	p12	210	203	7.622	8.286	-3.955	0	0.001
Nigeria	p13	210	203	7.216	7.975	-4.227	0	0
Nigeria	p14	210	203	7.524	8.31	-4.529	0	0
Nigeria	p15	210	203	7.699	8.345	-3.677	0	0.002
Nigeria	p16	210	203	8.351	8.673	-1.824	0.069	0.166
Nigeria	p17	210	203	8.033	8.655	-3.907	0	0.001
Nigeria	p18	210	203	7.928	8.302	-2.201	0.028	0.078
Nigeria	p19	210	203	7.584	8	-2.434	0.015	0.05
Nigeria	p20	210	203	7.629	7.851	-1.38	0.168	0.317
Nigeria	p21	210	203	7.856	8.188	-1.92	0.056	0.142
Nigeria	p22	210	203	7.962	8	-0.213	0.831	0.926
Nigeria	p23	210	203	7.5	8.207	-3.952	0	0.001
Nigeria	p24	210	203	6.77	7.76	-4.897	0	0
Nigeria	p25	210	203	7.867	8.404	-3.034	0.003	0.011
Nigeria	p26	210	203	7.433	7.837	-2.412	0.016	0.051
Nigeria	p27	210	203	7.053	7.627	-3.147	0.002	0.008
Nigeria	p28	210	203	6.986	7.667	-3.484	0.001	0.004
Nigeria	p29	210	203	7.274	8.015	-4.216	0	0
Nigeria	p30	210	203	7.385	7.915	-2.935	0.004	0.015
Nigeria	p31	210	203	6.626	7.41	-3.528	0	0.003
Nigeria	p32	210	203	7.337	7.921	-3.348	0.001	0.005
Nigeria	p33	210	203	7.833	8.379	-3.295	0.001	0.005
Nigeria	p34	210	203	7.59	8.276	-4.375	0	0
Nigeria	p35	210	203	7.519	8.084	-3.411	0.001	0.004
Nigeria	p36	210	203	7.469	7.837	-2.124	0.034	0.093
Nigeria	p37	210	203	7.21	8.153	-5.106	0	0
Nigeria	p38	210	203	7.082	7.495	-2.028	0.043	0.115
Nigeria	p39	210	203	6.883	7.419	-2.463	0.014	0.046
Nigeria	p40	210	203	6.805	7.62	-4.332	0	0
Nigeria	p41	210	203	7.172	8.054	-5.132	0	0
Nigeria	p42	210	203	7.595	8.069	-2.88	0.004	0.017
Nigeria	p43	210	203	7.584	8.005	-2.319	0.021	0.062
Nigeria	p44	210	203	7.428	7.841	-2.494	0.013	0.044
Nigeria	p45	210	203	7.177	7.79	-3.535	0	0.003
Tanzania	p01	206	185	9.146	8.173	5.318	0	0
Tanzania	p02	206	185	8.771	7.876	4.201	0	0
Tanzania	p03	206	185	7.965	8.027	-0.226	0.821	0.92
Tanzania	p04	206	185	6.736	6.071	1.843	0.066	0.162
Tanzania	p05	206	185	7.692	7.73	-0.137	0.891	0.97
Tanzania	p06	206	185	5.827	6.168	-0.923	0.357	0.547
Tanzania	p07	206	185	8.5	8.039	1.901	0.058	0.146
Tanzania	p08	206	185	8.292	7.549	2.984	0.003	0.013
Tanzania	p09	206	185	7.765	7.827	-0.21	0.834	0.926
Tanzania	p10	206	185	9.02	8.038	5.236	0	0
Tanzania	p11	206	185	9.064	8.097	5.329	0	0
Tanzania	p12	206	185	9.01	7.995	5.173	0	0
Tanzania	p13	206	185	8.626	8.032	2.886	0.004	0.017
Tanzania	p14	206	185	8.777	7.956	4.082	0	0.001
Tanzania	p15	206	185	8.99	8.272	3.656	0	0.002
Tanzania	p16	206	185	9.185	8.357	4.483	0	0
Tanzania	p17	206	185	8.941	8.103	4.155	0	0
Tanzania	p18	206	185	9.108	7.842	5.928	0	0
Tanzania	p19	206	185	8.789	7.95	3.867	0	0.001
Tanzania	p20	206	185	8.852	7.579	5.6	0	0
Tanzania	p21	206	185	8.736	8.059	3.303	0.001	0.005
Tanzania	p22	206	185	8.754	8.027	3.47	0.001	0.004
Tanzania	p23	206	185	9.025	7.989	5.15	0	0
Tanzania	p24	206	185	8.354	7.503	3.391	0.001	0.004
Tanzania	p25	206	185	8.762	8.295	2.308	0.022	0.062
Tanzania	p26	206	185	8.387	8.065	1.441	0.15	0.299
Tanzania	p27	206	185	8.128	7.743	1.631	0.104	0.229
Tanzania	p28	206	185	8.106	7.558	2.214	0.027	0.076
Tanzania	p29	206	185	8.168	7.541	2.499	0.013	0.043
Tanzania	p30	206	185	8.541	7.636	3.863	0	0.001
Tanzania	p31	206	185	7.608	7.114	1.78	0.076	0.181
Tanzania	p32	206	185	8.803	8.12	3.397	0.001	0.004
Tanzania	p33	206	185	8.932	8.151	3.902	0	0.001
Tanzania	p34	206	185	8.877	8.2	3.414	0.001	0.004
Tanzania	p35	206	185	8.545	8.049	2.352	0.019	0.058
Tanzania	p36	206	185	8.562	8.017	2.56	0.011	0.039
Tanzania	p37	206	185	8.835	8.13	3.406	0.001	0.004
Tanzania	p38	206	185	8.277	7.566	3.088	0.002	0.01
Tanzania	p39	206	185	7.819	7.797	0.09	0.928	0.977
Tanzania	p40	206	185	8.547	7.115	5.966	0	0
Tanzania	p41	206	185	8.824	7.918	4.386	0	0
Tanzania	p42	206	185	8.467	7.908	2.552	0.011	0.039
Tanzania	p43	206	185	8.747	7.876	4.387	0	0
Tanzania	p44	206	185	8.264	7.725	2.191	0.029	0.079
Tanzania	p45	206	185	8.454	7.875	2.641	0.009	0.033

## Discussion

When citizens from six middle-income countries had the opportunity to deliberate policy proposals relating to antibiotic resistance armed with evidence-based considerations and the ability to weigh competing pros and cons, there was a high level of support for evidence-based proposals that could impact their lives considerably. The average level of collective support after deliberation for all proposals combined was >70%, (>90% for a selected two-thirds of the proposals), and the deliberative process led to increased support for 95% of proposals. The largest increase in support was in two countries that showed the lowest support before deliberation - Tanzania and Indonesia.

Infection prevention proposals were most highly supported. All six countries have high burdens of infection with variable access to WASH, childhood vaccination, and other prevention programmes. High levels of poverty, poor nutrition, and immunosuppressed populations further increase vulnerability to pathogens, and these factors may have driven the popularity of infection prevention proposals. However significant increase in support following deliberation by Tanzanian participants suggests that the deliberative process itself can change perspectives. Support for infection prevention interventions will be critical to mitigating antibiotic resistance and were an area of focus for commitments adopted by the political declaration arising from the UNGA HLM-AMR in 2024. The importance of public buy-in will be a key factor in driving the behaviour change that will be needed to accomplish infection prevention and other interventions. Awareness and education proposals that are common subjects of national awareness programmes are highly relatable for the public and may explain in part their popularity with participants.

We observed differences in support for proposals relating to access to antibiotics and the use of antibiotics in food production, between LATAM (particularly Brazil) and African/Asian country participants. Weaker health systems in Africa and Asia and challenges in access to health care workers leads to increased reliance on informal antibiotic sellers such as retail pharmacists, medicine vendors, and the allied veterinarian workforce for animals. This may explain the greater support for informal sellers to be able to increase access of citizens to antibiotics in African and Asian countries. Increased access to antibiotics could have a major impact on the close to 3 million people dying each year of bacterial sepsis due to antibiotic-sensitive bacterial infections. ^1^ Relative opposition to proposals restricting antibiotics in the food chain from Brazilian and Colombian participants may relate to the importance of meat production as an industry in these countries in relation to export markets and the political economy.

Effective and equal participation is a defining criterion of functional democracies. Having equal opportunities to understand issues, their pros and cons, and having information on means, ends, and their potential impact are important drivers of participation. ^16^ Knowledge regarding antibiotic resistance increased during this study and the fact that the education level of participants itself was not a consistent factor in higher rating of policy proposals prior to deliberation, suggests that the deliberation process could be valuable in raising knowledge for those at any education level. Political efficacy too is a critical driver of political participation by citizens, ^17^ hence the fact that deliberation increased internal political efficacy i.e., participants’ perception of their influence on public policy and ability to create changes in their government, is important.

Several deliberative methodologies have been used to understand citizens’ views on antimicrobial resistance. Over the last decade, citizens’ juries in Wales, ^18^ England, ^19^ and Malawi ^20^ have deliberated AMR. Their inclusivity ^21^ i.e., the representativeness of a citizens’ jury varies; a systematic review of citizens’ juries for health policy decision making, found that although most studies were explicit in their stated aim of recruiting a jury that was descriptively representative of the community, the extent to which recruitment strategies succeeded in creating an inclusive environment varied considerably. ^11^ The number of jurors in the Wales, English, and Malawi juries ranged between 14–18, small numbers that also challenge inclusivity. Unlike Deliberative Polling ^®^, citizens’ juries aim to reach consensus opinion on positions to the questions or proposals being deliberated. This may reduce the importance of individuals’ views in favour of the ability to reach a common ground.

Citizens’ assemblies are larger than their jury counterparts (50–150 people) and meet over a series of timepoints to deliberate to help develop and evaluate policy. ^22^ Like juries, they orient towards consensus, leading to potential bias from the social pressures to get agreement, which has brought criticism. Rather than consensus, Machin highlights the importance of disagreement from within and outside politics for a ‘democratic, engaging, passionate, creative, and representative sustainability politics’. ^23^ Furthermore, an analysis of citizens’ assemblies in Ireland, a country where they have played a prominent role, has highlighted that citizens who support and participate in assemblies tend to be those who disagree or are dissatisfied with government policies or who are more politically engaged. ^12^ Those dissatisfied, tend to support political reforms, whereas those politically active, are driven towards deliberative modes of politics. No attitudinal data is collected in most citizens assemblies at the start (Ireland and France for example). If we do not know if the sample is representative at the start, that challenges the call to pay attention to the results at the end. Furthermore neither the citizens assemblies nor the citizens juries tend to have control groups for comparison, either for the attitudinal representativeness or for the opinion changes (difference in differences). It is much harder to draw inferences from such data about what the public would really want to do, if they were somehow engaged to think about the issues in depth and in an evidence-based environment.

Responsive dialogues engaging small groups of citizens in deliberation have also been applied to antibiotic-resistant infections. ^24^ The methodology is generally developed over a longer time frame to enable greater stakeholder engagement. An important difference from Deliberative Polling ^®^ is a predominant focus on co-creation of local solutions. Several responsive dialogues on antibiotic resistance are underway including in Thailand, ^25^ and South Africa. ^26^ However the small size of the samples in responsive dialogues obviously raises questions about representativeness, just like the Citizens Juries. Can such a small group speak for the nation?

The strengths of the Deliberative Polling ^®^ method is that, unlike juries and assemblies, Deliberative Polls are less likely to suffer from the social comparison effect that produces conformity, as final judgements are collected confidentially. ^13^ We ran this study fully online, increasing ease of access for participants. Stanford’s AI-assisted deliberation platform, encourages participation by all deliberants, through a series of nudges to share views and partake in the small group discussions, and can be easily scaled up to increase participant numbers. Our sample size of 400 per country is larger than citizens juries or assemblies and participants were recruited in a randomised fashion using accurate population data to determine benchmarks for critical demographic variables, such as gender, age, and domicile. This increases representativeness and inclusivity. Our choice of six countries on three different southern hemisphere continents brings a diversity of context and citizens albeit who share a common burden of bacterial infections, antibiotic use, and antibiotic resistance.

Our study has several limitations. Our focus on middle-income countries does not provide insights into the opinions of the public from low- or high-income countries, the unrepresented continents of Europe, North America, and Australasia. Second, the number of participants in each country and the number of countries in our study was constrained by budget. Whilst we recruited expert panellists from the six study countries for the plenary sessions and the co-authors have experience of working with clinicians and researchers from the included countries, none of the co-authors were from the countries themselves, which may have limited the nuance of interpretation of our findings. While the numbers were large enough to evaluate the representativeness via comparisons to the control group at time 1 (and other data) and large enough to evaluate the significance of opinion changes (including Difference in Differences in comparison to the pre-post control group) limited number of participants is a constraint on the extent to which sub-populations can be evaluated. Thirdly, although deliberation significantly increased overall support for proposals, given that many proposals were highly rated before deliberation, there might have been ceiling effects that prevented support going significantly higher, thus limiting the impact of the deliberation on changes in a selection of proposal ratings.

This is the first time that Deliberative Polling ^®^ has been used to engage citizens on the topic of antibiotic resistance. When given the chance to deliberate in small group discussions and plenary sessions over two days, citizens of 6 middle-income countries sharing a high burden of antibiotic resistance overwhelmingly supported proposals that could substantially affect their lives. These proposals were chosen to represent the likely policy commitments to be supported by member states in the political declaration arising from UNGA HLM-AMR24, and were indeed, heavily represented in the final political declaration adopted by member states, particularly those relating to access to antibiotics, diagnostics and infection prevention tools (WASH, vaccines etc), all of which were highly supported by our participating country groups. The deliberation process itself, increased support for proposals, knowledge on antibiotic resistance and the internal political efficacy of participants. Government policy directly affects the public and it is the public who must change behaviour to enact policies such as those to mitigate antibiotic resistance. Deliberative Polling ^®^ allows the voice of the people to be heard. For example, since the first Deliberative Polling ^®^ project was undertaken in Mongolia in 2015, a law was passed that requires Deliberative Polling ^®^ on potential amendments to the Mongolian Constitution before they are considered by parliament. ^27^ As a means of community engagement, it provides bidirectional benefit to policymakers and participants on a potentially large scale: the public have their voices heard about topics that directly affect them, gain knowledge, and internal political efficacy; governments, ensure that the voice of the people is heard augmenting the democratic process, and allowing for alterations to policies and laws.

Efforts to mitigate the antibiotic resistance pandemic would benefit greatly from wider participation and co-development of policies between governments and the public, who are too often forgotten in favour of a top-down approach. As the main governance body for AMR, we advise the Quadripartite Joint Secretariat on AMR to consider building on our work by working with the research community to use Deliberative Polling ^®^ Methodology for a global poll.

## Data Availability

All data underlying the findings of this study are openly available in the Stanford Digital Repository under the title ‘Deliberative Poll on Antimicrobial Resistance (AMR) - Harmonized dataset’ at https://doi.org/10.25740/sb639ms2957. ^28^ The dataset includes the survey dataset and extended data accompanied by comprehensive codebooks. This project contains the following underlying data:
1. README.md (Instructions on content of the folder) 2. Extended Data a. Survey questions asked to participants pre and post deliberation b. Forty five policy proposals considered by the Intervention Group 3.
AMR_harmonized_codebook.pdf (Codebook with explanation of variables). 4.
AMR_harmonized_numeric.csv (All countries dataset in CSV format). 5.
AMR_harmonized_codebook.xlsx (All countries codebook to explain variables in XLSX format). Data is available under the terms of the CC BY 4.0 license.
