# Supplementary material for: Understanding what citizens think about Antimicrobial Resistance: Deliberative Polling® in six middle-income countries
Source: Wellcome Open Res. 2026 May 14;10:690. Originally published 2025 Dec 22. [Version 2] doi: 10.12688/wellcomeopenres.24803.2 (PMC12891964; doi:10.12688/wellcomeopenres.24803.2)
Supplement: Peer review history

# Reviewer report: Reviewer response for version 2

**DOI:** 10.21956/wellcomeopenres.29023.r155583

**Published:** 2026-05-21

**Authors:** Martin Mickelsson

**Affiliations:** 1 University of Gothenburg, Gothenburg, Västra Götaland County, Sweden

I have looked over the revised version and while there have been some restructuring and clarifications, I view it that my comments for the most part stand. I would ask the authors to clarify these points in order for the paper to realize its potential. I would give this article the same status as last round, **Approved with Reservations.  **I would suggest the authors address the points I raised to realise the potential of the paper.

 The most pressing to my mind are comment 3 and 4.

 Comment 3. Ethical approval and study country review

 The manuscript reports ethical approval from Stanford university institutional Review Board. Meanwhile, the study involved community participants in six middle-income countries. It is not quite unclear if any formal ethical approval was sought in the respective study countries. I would suggest the authors clarify if ethical approval was sought or required in each participating country. In the case no national ethical approval in the study countries was sought, how was this determination made and where contextual risk of this choice evaluated. This could potentially be addressed by specifying if local collaborators in the study countries were involved in assessing regulatory requirements. This clarification would be especially valuable considering the global north- global south character of the research.

 Comment 4. Structure of the Discussion Section

 The discussion section contains extensive descriptions of alternative deliberative methodologies, including citizen juries and citizen assemblies. While this contextualization is valuable, it appears misplaced in the discussion section. These methodological comparisons would be more appropriate in the methods section. The insights gathered from the analysis of the results would be better served if the discussion section focused on presenting the interesting interpretation results and situating the study within broader theoretical debates.

Is the work clearly and accurately presented and does it cite the current literature?

Partly

If applicable, is the statistical analysis and its interpretation appropriate?

Yes

Are all the source data underlying the results available to ensure full reproducibility?

Yes

Is the study design appropriate and is the work technically sound?

Yes

Are the conclusions drawn adequately supported by the results?

Yes

Are sufficient details of methods and analysis provided to allow replication by others?

Yes

Reviewer Expertise:

NA

I confirm that I have read this submission and believe that I have an appropriate level of expertise to confirm that it is of an acceptable scientific standard, however I have significant reservations, as outlined above.

# Reviewer report: Reviewer response for version 2

**DOI:** 10.21956/wellcomeopenres.29023.r155587

**Published:** 2026-05-20

**Authors:** Lindsey A Edwards

**Affiliations:** 1 King’s College London, London, England, UK

I have no further comments to make. I believe the authors have appropriately addressed the points raised in the previous review and have made the necessary revisions to strengthen the manuscript. I would like to congratulate the authors on this highly informative and well-executed study.

Is the work clearly and accurately presented and does it cite the current literature?

Yes

If applicable, is the statistical analysis and its interpretation appropriate?

Yes

Are all the source data underlying the results available to ensure full reproducibility?

Yes

Is the study design appropriate and is the work technically sound?

Yes

Are the conclusions drawn adequately supported by the results?

Yes

Are sufficient details of methods and analysis provided to allow replication by others?

Yes

Reviewer Expertise:

Antimicrobial resistance; Microbiome; host-microbial interactions; mucosal immunology; AMR Policy and Microbial-based product regulation.

I confirm that I have read this submission and believe that I have an appropriate level of expertise to confirm that it is of an acceptable scientific standard.

# Reviewer report: Reviewer response for version 1

**DOI:** 10.21956/wellcomeopenres.27326.r146666

**Published:** 2026-03-04

**Authors:** Martin Mickelsson

**Affiliations:** 1 University of Gothenburg, Gothenburg, Västra Götaland County, Sweden

Summary

 This paper engages with the key sustainable health challenge of AMR and through the use of a scalable deliberative methodology is able to engage with communities perspectives across global regions such as Africa, Latin America and Asia. The paper has great potential in making a meaningful contribution to perspectives on AMR policy and governance and the need to consider public support and engagement for these global efforts to be successful. Combining knowledge and deliberative capacity-building with opinion -polling presents an exciting avenue of research, moving beyond current methodological limitations. The paper also clearly shows changes in views of the public to AMR policies as well as national and regional difference in the resulting data. 

 Assessment

 Overall, the paper presents a compelling account of public views and approaches to key AMR policy initiatives and also insights in regarding the importance of knowledge and capacity-building along with opinion polling. The results are likely to make a significant contribution to the field.

 Meanwhile, I have four primary comments that I would suggest the authors address in order for the paper to realise its potential.

 Comment 1. Theoretical framing and recommendation to engage with deliberative democratic theory

 The methodology is described as building on deliberative democratic theory. However, engagement with the theory is limited to brief mention in the abstract, with minimal discussion in the main body of the manuscript. To this end, I would suggest the authors included a brief motivation for grounding the methodology in deliberative democratic theory. This would also help the reader to understand how the theory informed research design and analysis. I would also recommend some engagement with key critiques of deliberative democratic theory. For example has the theory been criticized for possible limitations of its global north origins, particularly when operationalised in research with communities in the global south. Addressing some of these critiques in relation to the study would strengthen the conceptual basis of the paper.

 Comment 2. Country selection and inclusion criteria

 In the introduction and limitations section it is stated that including countries with different socioeconomic resources would provide a clearer global picture. Meanwhile, the study focuses on middle-income countries. It would be helpful if this choice was explained a bit clearer. Also a short note on how this choice affected the generalizability of the results.

 Comment 3. Ethical approval and study country review

 The manuscript reports ethical approval from Stanford university institutional Review Board. Meanwhile, the study involved community participants in six middle-income countries. It is not quite unclear if any formal ethical approval was sought in the respective study countries. I would suggest the authors clarify if ethical approval was sought or required in each participating country. In the case no national ethical approval in the study countries was sought, how was this determination made and where contextual risk of this choice evaluated. This could potentially be addressed by specifying if local collaborators in the study countries were involved in assessing regulatory requirements. This clarification would be especially valuable considering the global north- global south character of the research.

 Comment 4. Structure of the Discussion Section

 The discussion section contains extensive descriptions of alternative deliberative methodologies, including citizen juries and citizen assemblies. While this contextualization is valuable, it appears misplaced in the discussion section. These methodological comparisons would be more appropriate in the methods section. The insights gathered from the analysis of the results would be better served if the discussion section focused on presenting the interesting interpretation results and situating the study within broader theoretical debates.

 In conclusion

 The paper addresses a timely and essential global challenge in a exciting new way with the use of an deliberative approach. Addressing the points raised would significantly strengthen the paper and allow it to make a significant contribution to the field.

Is the work clearly and accurately presented and does it cite the current literature?

Partly

If applicable, is the statistical analysis and its interpretation appropriate?

Yes

Are all the source data underlying the results available to ensure full reproducibility?

Yes

Is the study design appropriate and is the work technically sound?

Yes

Are the conclusions drawn adequately supported by the results?

Yes

Are sufficient details of methods and analysis provided to allow replication by others?

Yes

Reviewer Expertise:

Antimicrobial resistance research, health literacy research, socio-ecological health research, One health research

I confirm that I have read this submission and believe that I have an appropriate level of expertise to confirm that it is of an acceptable scientific standard, however I have significant reservations, as outlined above.

# Reviewer report: Reviewer response for version 1

**DOI:** 10.21956/wellcomeopenres.27326.r146667

**Published:** 2026-02-24

**Authors:** Lewis Husain

**Affiliations:** 1 Institute of Development Studies, Brighton, England, UK

This is an interesting and novel approach to understanding population attitudes to certain options for dealing with AMR.

 The article is methodologically sound and provides a comparison, in the discussion, between the method used here – deliberative polling – and other potentially functionally similar methods, including citizens juries, etc.

 These seems like a useful addition to the methods available to study policy preferences towards this issue, but with some potential questions or caveats:

 1. It is puzzling that the deliberative polling found high levels of support for most policy proposals at baseline. This is mentioned in the article (“Given that so many proposals were highly rated before deliberation …”) but not fully explored.

 Given that the received wisdom is that there are generally low levels of awareness of AMR in most populations, and Figure 4 shows mostly low to medium levels of prior understanding of AMR, why are we seeing high levels of support, pre-intervention?

 Does this indicate rationales for supporting some/many of these policy proposals on the part of both control and treatment groups that are unrelated to AMR – for example, many of the proposals in Table 8 are likely to be universally popular, whether or not an individual is concerned about AMR (though in contrast many related to equity, access, and stewardship are quite focused on antibiotics)?

 It’s not obvious that there’s a clear-cut difference between support to more general measures (e.g. better infection prevention, clean water) and to ones specifically related to antibiotics/AMR (e.g. increasing access, reporting use). This is puzzling, given the levels of prior awareness of AMR.

 How do the authors understand this high-level prior support of appropriate priorities despite the low (Tanzania) to medium (India, Indonesia, Colombia) prior understanding of most populations of AMR (Figure 4)?

 2. It is unclear that this methodology can be replicated at scale, given its time- and resource-intensive nature. If this is the case, then it seems its main use is as a barometer of *informed* public support. From a policy/political perspective, this then seems to create two questions:

 a. What do the high pre- and post- numbers fundamentally mean? That there are existing high levels of popular support for many of measures to tackle AMR, and that there is a substantial (c. 7-10%) gain from this kind of knowledge intervention, but one that is unlikely to be determinative of political support?

 b. What kinds of population-based knowledge interventions could be used to achieve similar effects, rather than at the relatively small scale of this kind of intervention?

 3. It seems that costs and trade-offs were not included in the rankings of specific policy proposals. For example, extending WASH at scale is likely to be universally popular, but comes with a high cost. Questions such as comparative rankings and willingness to pay for certain actions is politically important information and could potentially be included in future work.

 It would be helpful to see more discussion of these issues in the final version.

Is the work clearly and accurately presented and does it cite the current literature?

Yes

If applicable, is the statistical analysis and its interpretation appropriate?

I cannot comment. A qualified statistician is required.

Are all the source data underlying the results available to ensure full reproducibility?

Yes

Is the study design appropriate and is the work technically sound?

Yes

Are the conclusions drawn adequately supported by the results?

Yes

Are sufficient details of methods and analysis provided to allow replication by others?

Yes

Reviewer Expertise:

Relevant to this article: Health systems, AMR related policy, with a focus on R&D, innovation and global health.

I confirm that I have read this submission and believe that I have an appropriate level of expertise to confirm that it is of an acceptable scientific standard, however I have significant reservations, as outlined above.

# Reviewer report: Reviewer response for version 1

**DOI:** 10.21956/wellcomeopenres.27326.r143137

**Published:** 2026-02-10

**Authors:** Amy Pruden

**Affiliations:** 1 Virginia Tech, Blacksburg, VA, USA

Here the authors survey public support for interventions aimed at curbing the evolution and spread of antibiotic resistance and the effects of various modes of public education.  The study is timely and important given the United Nations General Assembly high level meeting on AMR in 2024 and commitments to action that require public buy-in. The method is innovative relative to typical surveys and education campaigns, employing "Deliberative Polling," which is built on principles of democratic process and probes individual participants' dispositions after extended evidence-based discussions. The authors provide citations to justify the method as appropriate, given that alternative methods often harvest "ghost" opinions or unintentionally bias the participants' responses. The insights gained from the approach are impressive and I believe will be highly valuable towards advancing effective education and policy measures to stem the spread of AMR.

 The authors focused in particular on six middle income countries (MICs) across three continents, which provided a number of insights into existing disposition towards AMR policy measures, including contrasts according to gender and rural/urban residence of participants. Some of the same proposed measures tended to rise to the top across all countries, while there was a bit more push-back found for certain measures in specific countries (e.g., reducing the role of pharmacists in dispensing drugs in South America).  I thought that it was especially encouraging to see how improving infection control and basic water sanitation and hygiene (WASH) conditions and their connection to AMR were readily understandable to the general populace. Overall, the authors were thorough in making all of the rich data gathered from this study accessible, while also distilling down some key overarching observations in digestible figures and tables for a general audience.  It will be very valuable to publish this study in an open access form, as these data will be very valuable globally as more and more nations operationalize their national action plans for combating AMR.

 I have only a few minor suggestions for the authors to double check and address to improve the manuscript.

 The logic of the phrasing of the Conclusion is a bit difficult to follow "and this is now for AMR helping us to understand..."

 second to last paragraph of plain language summary- the word "also" does not have a logical connection to the previous two sentences.

 last sentence of plain language summary: It's not entirely clear if the six countries had "different socioeconomic resources"- or if they were considered the same for the purpose of this study and thus this is a recommendation for future studies?  It seems to me that just because countries are classified as MICs, doesn't mean that their socioeconomic resources are the same?

 under Methods- there appears to be a grammar issue with the second half of the sentence "is a global public opinion company" not matching the first in verb number. Also, if the authors choose to use the acronym RDR- they should define it in the first instance and use it consistently thereafter

 Methods- you could be a little more precise and say about census data- not only that it is accurate, but high resolution "they used accurate and high resolution population data"

 under Surveys and proposals- it would be helpful to have an better idea of how these 45 proposals were landed upon?  Some seem potentially counterproductive to the primary goal of decreasing antibiotic resistance, though they do address larger issues such as access to appropriate medical care and pharmaceuticals.

 Was it intended that all 45 proposals are "good" proposals, i.e., helpful to curb AMR, or are some intentionally counterproductive?

 Table 1 proposal 1, is there a typo, or is this the verbatim proposal that was offered to participants- it should be "ensure appropriate access to antibiotics" not "accessed"-- There are a few typos and syntax errors in this table that I suggest that the authors scan for--then again, given that the survey was multi-lingual, maybe this is not critical.

 Speaking of which- I wonder if there were any language barriers to this study- or examples of things that translate differently?  Was this not an issue?

 Results- it is especially exciting to see citizen support for improvement in WASH as an AMR mitigating measure- given that there are broad co-benefits, yet it's a little more challenging conceptually to explain to the general populace the connection between WASH and AMR

 Opening paragraph of Discussion- again, I am a bit unclear about how aware or educated participants were about trade-offs that could personally impact them, as implied by the statement "there was a high level of support for evidence-based proposals that could impact their lives considerably."

 Discussion- limitations-- This study seems an interesting and exemplary example of AI-assisted research.  Were there not any obstacles, pitfalls, limitations, etc.- that resulted from an AI-assisted approach to interacting with participants? This is important in terms of advice and considerations for future studies.

Is the work clearly and accurately presented and does it cite the current literature?

Yes

If applicable, is the statistical analysis and its interpretation appropriate?

Yes

Are all the source data underlying the results available to ensure full reproducibility?

Yes

Is the study design appropriate and is the work technically sound?

Yes

Are the conclusions drawn adequately supported by the results?

Yes

Are sufficient details of methods and analysis provided to allow replication by others?

Yes

Reviewer Expertise:

Anthropogenic drivers of AMR in the environment (wastewater, surface water, drinking water), microbial ecology of AMR, and effect of engineered interventions (agricultural, wastewater) of environmental sources to mitigate the spread of AMR and reduce probability/risk of humans acquiring resistant infections.

I confirm that I have read this submission and believe that I have an appropriate level of expertise to confirm that it is of an acceptable scientific standard.

# Response

**Published:** 2026-03-23

**Authors:** Louise Gough

**Affiliations:** The Trinity Challenge, Cambridge, England, UK

Many thanks for your kind comments and for reviewing our publication. We are much indebted to you for helping to augment our message.

**Point 1- The logic of the phrasing of the Conclusion is a bit difficult to follow "and this is now for AMR helping us to understand...**

**Response: **Thank you, we will rectify in the next opportunity to review the introductory sections.

**Point 2- second to last paragraph of plain language summary- the word "also" does not have a logical connection to the previous two sentences. ‘Also’ links the effect of DP in the first sentence with its effect on knowledge and political efficacy.**

**Response: **Thank you, we plan to rectify with - *There was a high level of collective support for the majority of proposals, which increased through the deliberative process. Antibiotic access and food security proposals showed regional variation in support. Deliberation also increased knowledge about antibiotic resistance and participants’ perception of their influence on public policy and ability to create changes in their government.*  

**Point 3- last sentence of plain language summary: It's not entirely clear if the six countries had "different socioeconomic resources"- or if they were considered the same for the purpose of this study and thus this is a recommendation for future studies?  It seems to me that just because countries are classified as MICs, doesn't mean that their socioeconomic resources are the same?**

**Response: **We agree that different country-level classifications each have their deficiencies, but using income status is the best option. We have modified the sentence to read: *‘Polling high- and low-income countries would give an expanded picture across different resource settings’*. We have also added a new Table in relation to a point raised by another reviewer, to give a better framing of each country demographics and AMR levels.  

**Point 4- under Methods- there appears to be a grammar issue with the second half of the sentence "is a global public opinion company" not matching the first in verb number.**

**Response: **Thank you, we will rectify in the next opportunity with *‘are global public opinion companies’ *  

**Point 5- Also, if the authors choose to use the acronym RDR- they should define it in the first instance and use it consistently thereafter.**

**Response: **Thank you. Rectified. 

**Point 6- Methods- you could be a little more precise and say about census data- not only that it is accurate, but high resolution "they used accurate and high resolution population data"** Thank you. Rectified. **Point 7- under Surveys and proposals- it would be helpful to have an better idea of how these 45 proposals were landed upon?  Some seem potentially counterproductive to the primary goal of decreasing antibiotic resistance, though they do address larger issues such as access to appropriate medical care and pharmaceuticals. Was it intended that all 45 proposals are "good" proposals, i.e., helpful to curb AMR, or are some intentionally counterproductive?**

**Response: **As you point out, some proposals were chosen to help participants deliberate on questions of equity and access in countries where informal access to antibiotics is widespread. This is in line with the UN political declaration’s commitment to “ *leave no one behind and reach the furthest behind first”*

**Point 8- Table 1 proposal 1, is there a typo, or is this the verbatim proposal that was offered to participants- it should be "ensure appropriate access to antibiotics" not "accessed"-- There are a few typos and syntax errors in this table that I suggest that the authors scan for--then again, given that the survey was multi-lingual, maybe this is not critical.**

**Response: **Thank you. Rectified.  

**Point 9- Speaking of which- I wonder if there were any language barriers to this study- or examples of things that translate differently?  Was this not an issue?**

**Response: **One can never say 100%, but simultaneous translation was available in the main languages of the countries involved during the small group and plenary sessions, as referred to in the text.

**Point 10- Results- it is especially exciting to see citizen support for improvement in WASH as an AMR mitigating measure- given that there are broad co-benefits, yet it's a little more challenging conceptually to explain to the general populace the connection between WASH and AMR.**

**Response: **Agreed.

**Point 11- Opening paragraph of Discussion- again, I am a bit unclear about how aware or educated participants were about trade-offs that could personally impact them, as implied by the statement "there was a high level of support for evidence-based proposals that could impact their lives considerably."**

**Response: **The aim of the deliberative process was to make participants aware and educate them about exactly that i.e., the trade-offs of each proposal.   

**Point 12- Discussion- limitations-- This study seems an interesting and exemplary example of AI-assisted research.  Were there not any obstacles, pitfalls, limitations, etc.- that resulted from an AI-assisted approach to interacting with participants? This is important in terms of advice and considerations for future studies.**

**Response: **We are always learning from not only the findings of the deliberative platform, but the participants' experiences and evaluations of the platform. From the participants' experience, since the deliberation platform is a video-based platform, there is always a chance there are technical problems that occur. Over time, our technical team has developed easy ways for participants to resolve the vast majority of technical issues that may occur. For example, the most typical technical problems are not being able to hear or use the microphone. These are often browser or computer setting issues and the platform now has easy ways to overcome these problems. But, as much as we try to anticipate all the possible technical problems, there are always a couple of participants from time to time that have technical problems that delay their participation. We are always thinking about how to improve the participants' experience, especially in these situations when participants are waiting to resolve their issues.  In terms of during deliberations, our goal is to provide a deliberative experience that closely mimics an in-person deliberation for the participants. For example, when the platform nudges participants, the platform draws suggestions for the nudges from the briefing materials in the agenda. We do not employ LLMs to 'listen' to the conversations, so the nudges may not always be completely in line with the current discussion. As we continue to conduct experiments to better understand the impacts of certain technologies, we aim to provide participants a deliberative experience that closely matches what an in-person deliberation would be like. Thank you for taking the time to review our paper. 

# Reviewer report: Reviewer response for version 1

**DOI:** 10.21956/wellcomeopenres.27326.r143134

**Published:** 2026-02-09

**Authors:** Emma Pitchforth

**Affiliations:** 1 Department of Health and Community Sciences, University of Exeter Medical School, Exeter, UK

**Summary**

 Tackling antimicrobial resistance requires global agreement and action but local implementation and buy-in. This study looks at how the views of citizens in countries can be brought into the policy dialogue. It uses deliberative polling methodology to assess attitudes in six middle-income countries. In a randomised design the intervention group took part in a deliberative process around 45 proposed policies/actions. This involved small group discussions and plenary sessions. Control group participants did not receive anything and both groups completed a pre- and post- deliberation survey. The findings showed that support significantly increased for around three quarters of proposal as well as knowledge about AMR and political efficacy. There were some differences in results between countries. The authors conclude that deliberative polling offers the potential for large scale community engagement and a means to understand the views of the public relating to policies, which has been lacking in AMR.

**Comments**

 I really enjoyed reading the paper and it is exciting to have robust research that could make a difference to how policy dialogues take place and a means that bridges the dual need for global action and agreement and locally prioritised measures. The paper is very accessible in the style it is written. There are a lot of tables but given that there are six countries, 45 policies and pre-and post- analysis I am not sure where they opportunities to reduce would be.

 While the paper is very clearly written and presented, I have some suggestions that may strengthen it further.

 1. Global North perspective There is an impression reading the paper that it has been written from a global north perspective.

 ‘Over the summer’ is used in the abstract – this would be better given as months as the months of summer differ between the northern and southern hemispheres.

 Authorship does not appear to include anyone from the six countries where data collection took place. It may not be possible to change this but this should be reflected on in the paper, perhaps particularly around the insights to the interpretation of the differences between countries.

 Ethical approval was sought from Stanford but not in the countries where research was taking place. Many journals would require that ethical approval and necessary permissions had been sought in country too. It would be helpful if the authors would clarify if any steps were taken to do this or give an explanation as to why this was not done.

 The discussion says that ‘Our focus on middle-income countries across the southern hemisphere…’ . India is in the northern hemisphere and I think Indonesia spans northern and southern so it would be good just to increase the accuracy of this statement.

 Although Malawi is mentioned in some of the evidence brought into the discussion, there is a lot of reliance on examples of deliberative polling or approaches such as citizen juries in high income countries. It may be that these are most relevant to AMR but I would have liked so see more in the paper about the use of such approaches in the six countries that were involved in the country. This could be done in the contextual information provided (see comments below) or/and in the discussion.

 2. Selection of countries and contextual information

 I found the justification for the selection of the six studies, based on convenience and high rates of AMR, a bit weak or wanted to have more information about these countries. I think this would be valuable for a paper that is acknowledging the need to understand local priorities. I would encourage the authors to include more information on each country, perhaps in a table. Things that would be good to include would be, but there may be other factors too:

- Level of middle income country (upper/lower)
- History of deliberative democracy/polling
- Rates of AMR
- AMR policy context
- Governance (devolvement of power between national/state government)
- Main languages spoken
- Income levels

The authors speculate as to why there may be some of the differences found between countries in terms of support for different policies and this would be strengthened if more information is given in the paper. Overall, it feels as though more contextual information about the countries would help the paper.

 3. Discussion

 The discussion is clearly written. There is a lot of focus on deliberative polling versus other approaches. I felt I wanted a bit more about how these findings could be used by policymakers and why relevant for researchers – what would be the next steps? I also wondered if it was worth making more of the point that there are clear opportunities for addressing AMR through non-AMR specific policies. Because of the nature and scale of AMR as a research community we often frame AMR as an exceptional case while it seems to me some of the opportunities around AMR are through other, often existing, areas of policy. The paper highlights this around WASH, infection prevention, vaccination which are broader public health priorities, so if strengthened attitudes to this through deliberative processes, potential wins in multiple areas (but also perhaps helps explain high level of support at outset). How do we capitalize on such things?

 Finally, the paper concludes about the power of this method and the need to go to a global scale. I felt like I wanted more this at a bit more of practical level – who needs to do what and who needs to be involved for deliberative processes to make a difference? What are the priorities for research, beyond simply expansion? 

 More minor points:

1. In asking about gender in the survey (pg 6)could the authors clarify if this was a binary choice or not
2.  Could the authors make clear between what dates data collection took place in each of the countries (again this could be included in overview table)
3. Could the authors include date of IRB approval
4. Languages – could the authors give information on what percentage of populations in countries would have the language of tools as their first language. (e.g. in India, what percentage of population speak Hindi or English as first language?)
5. I wondered if the figure of the deliberative process could be adapted to show not only the stage of the deliberative process but both groups of the randomisation, this would make clear visually that controls were only doing poll 1 and 2.

Is the work clearly and accurately presented and does it cite the current literature?

Yes

If applicable, is the statistical analysis and its interpretation appropriate?

Yes

Are all the source data underlying the results available to ensure full reproducibility?

Yes

Is the study design appropriate and is the work technically sound?

Yes

Are the conclusions drawn adequately supported by the results?

Yes

Are sufficient details of methods and analysis provided to allow replication by others?

Yes

Reviewer Expertise:

AMR, health policy research, global health

I confirm that I have read this submission and believe that I have an appropriate level of expertise to confirm that it is of an acceptable scientific standard, however I have significant reservations, as outlined above.

# Response

**Published:** 2026-03-23

**Authors:** Louise Gough

**Affiliations:** The Trinity Challenge, Cambridge, England, UK

Thank you for your excellent review of our paper and the helpful suggestions that will augment its readability, interpretability, and impact. We have responded to your feedback below as follows: 

**Point 1- ‘Over the summer’ is used in the abstract – this would be better given as months as the months of summer differ between the northern and southern hemispheres.**

**Response: **Thank you for this observation. We have changed this to give the months that the study was conducted over.

**Point 2- Authorship does not appear to include anyone from the six countries where data collection took place. It may not be possible to change this but this should be reflected on in the paper, perhaps particularly around the insights to the interpretation of the differences between countries.**

**Response: **You make an excellent point. The lead author, Prof Marc Mendelson has over 20 years of experience working in LMICs, predominantly South Africa, so there is some LMIC representation, however, your point is well taken. Representatives from the study countries took part in all the expert plenary sessions during the deliberations. It is too late now to bring in other authors but we have added more clarity on these points in the discussion section. 

**Point 3- Ethical approval was sought from Stanford but not in the countries where research was taking place. Many journals would require that ethical approval and necessary permissions had been sought in country too. It would be helpful if the authors would clarify if any steps were taken to do this or give an explanation as to why this was not done.**

**Response: **The channel for ethical approval was taken considering previous multi-country Deliberative Polls, which required central approval from the originating academic institution i.e., Stanford University.

**Point 4- The discussion says that ‘Our focus on middle-income countries across the southern hemisphere…’ . India is in the northern hemisphere and I think Indonesia spans northern and southern so it would be good just to increase the accuracy of this statement.**

**Response: **Thank you. We have altered it to reflect this by deleting ‘ *across the southern hemisphere’*. 

**Point 5- Although Malawi is mentioned in some of the evidence brought into the discussion, there is a lot of reliance on examples of deliberative polling or approaches such as citizen juries in high income countries. It may be that these are most relevant to AMR but I would have liked so see more in the paper about the use of such approaches in the six countries that were involved in the country. This could be done in the contextual information provided (see comments below) or/and in the discussion.**

**Response: **The examples given do indeed refer mainly to AMR-related community engagement examples, although Malawi is not the only LMIC mentioned. Responsive dialogues in South Africa and Thailand are also given as examples. We felt that it was better to be more focused than go into the details of how DP has been used in many countries across the world. However, we have added the following text to the introduction:‘Deliberative Polling has been used in over 150 studies across over 50 countries' with the requisite reference. 

**Point 6- I found the justification for the selection of the six studies, based on convenience and high rates of AMR, a bit weak or wanted to have more information about these countries. I think this would be valuable for a paper that is acknowledging the need to understand local priorities. I would encourage the authors to include more information on each country, perhaps in a table.**

**Response: **Thank you. We have added a new Table to the methods section, including country-level demographics and AMR rates.

**Point 7- I felt I wanted a bit more about how these findings could be used by policymakers and why relevant for researchers – what would be the next steps? I also wondered if it was worth making more of the point that there are clear opportunities for addressing AMR through non-AMR specific policies. Because of the nature and scale of AMR as a research community we often frame AMR as an exceptional case while it seems to me some of the opportunities around AMR are through other, often existing, areas of policy. The paper highlights this around WASH, infection prevention, vaccination which are broader public health priorities, so if strengthened attitudes to this through deliberative processes, potential wins in multiple areas (but also perhaps helps explain high level of support at outset). How do we capitalize on such things? Finally, the paper concludes about the power of this method and the need to go to a global scale. I felt like I wanted more this at a bit more of practical level – who needs to do what and who needs to be involved for deliberative processes to make a difference? What are the priorities for research, beyond simply expansion?**

**Response: **Thank you for these interesting points. We have modified and added to, the last paragraph of the discussion as follows: *‘Government policy directly affects the public and it is the public who must change behaviour to enact policies such as those to mitigate antibiotic resistance. Deliberative Polling® is a powerful method of community engagement to understand the views of the public relating to the existential crisis of antibiotic resistance. Deliberative Polling® allows the voice of the people to be heard. For example, since the first Deliberative Polling® project was undertaken in Mongolia in 2015, a law was passed that requires Deliberative Polling® on potential amendments to the Mongolian Constitution before they are considered by parliament. As a means of community engagement, it provides bidirectional benefit to policymakers and participants on a potentially large scale: the public have their voices heard about topics that directly affect them, gain knowledge, and internal political efficacy; governments, ensure that the voice of the people is heard augmenting the democratic process, and allowing for alterations to policies and laws.* *Efforts to mitigate the antibiotic resistance pandemic would benefit greatly from wider participation and co-development of policies between governments and the public, who are too often forgotten in favour of a top-down approach. As the main governance body for AMR, we advise the Quadripartite Joint Secretariat on AMR to consider building on our work by working with the research community to use Deliberative Polling® Methodology for a global poll.’*

**Point 8- More minor points:**

**In asking about gender in the survey (pg 6)could the authors clarify if this was a binary choice or not** -

**Response: **Yes

**Could the authors make clear between what dates data collection took place in each of the countries (again this could be included in overview table)** -

**Response: **Complete, the pre- and post event questionnaires and deliberative events themselves all took place between June to August 2024. 

**Could the authors include date of IRB approval**

**Response: **Yes, now included

**Languages – could the authors give information on what percentage of populations in countries would have the language of tools as their first language. (e.g. in India, what percentage of population speak Hindi or English as first language?).**

**Response: **We do not believe that including this information will help in the interpretation of our findings as participants had the option of simultaneous translation into the main languages of the country during the deliberation process, as highlighted in the text.

**I wondered if the figure of the deliberative process could be adapted to show not only the stage of the deliberative process but both groups of the randomisation, this would make clear visually that controls were only doing poll 1 and 2.**

**Response: **We have made this clear in the text, so we would rather keep the figure as is. Thank you again for your time in reviewing this article.

# Reviewer report: Reviewer response for version 1

**DOI:** 10.21956/wellcomeopenres.27326.r143709

**Published:** 2026-01-22

**Authors:** Lindsey A Edwards

**Affiliations:** 1 King’s College London, London, England, UK

**Summary**

 Antimicrobial resistance (AMR) is a global, multisectoral crisis threatening health, food systems, the environment, and socioeconomic stability. Approximately 8 million sepsis‑related deaths occur annually, and antibiotics underpin survival from infection and enable modern medicine. Deaths from drug‑resistant infections are projected to reach 39 million over the next 25 years. The economic consequences are similarly severe, with major losses in livestock production and global GDP, and an estimated 24 million people at risk of being pushed into poverty by 2050.

 This study highlights that AMR interventions will require behaviour change and public cooperation, making citizen support essential for effective policy implementation. The authors investigate how members of the public in six middle‑income countries understand AMR and how they prioritise potential policy actions when given the opportunity to deliberate in depth. The work was timed to coincide with negotiations for the 2024 UN General Assembly High‑Level Meeting on AMR, aiming to bring informed citizen perspectives directly into global policy dialogue.

 The team used **Deliberative Polling®**, a method grounded in deliberative democratic theory, to explore informed public opinion at scale.

**Key features:**

- Conducted in **six middle‑income countries** across Africa, Asia, and South America.
- Participants were **randomised** to either:
  -
    - a deliberation group (receiving balanced written/video materials, facilitated small‑group discussions, and expert Q&A), or
    - a control group (survey only).
- Participants evaluated **45 policy proposals** aligned with the anticipated UN political declaration.

**Key Findings**

- **2419 participants** took part.
- After deliberation, support increased significantly for **three‑quarters** of the proposals.
- **Two‑thirds** of proposals reached **>90% support**.
- Knowledge about AMR and internal political efficacy also improved.
- **Infection prevention policies** received the strongest and most consistent support across all countries.
- Regional variation emerged for policies restricting informal antibiotic access and regulating antibiotic use in food production.

**General assessment**

 This manuscript addresses a critical global health challenge: antimicrobial resistance (AMR). The authors clearly set out the motivation for the study, noting that *“the pandemic of antimicrobial resistance (AMR) will only be mitigated by policy action and innovation and importantly, supported by local and community action”* and situates the work in the context of the 2024 UN General Assembly High‑Level Meeting on AMR. This framing effectively underscores the relevance of the study and provides a strong rationale for exploring citizens’ understanding and prioritisation of AMR‑related policies.

 The authors use Deliberative Polling® to engage citizens across six middle‑income countries, providing valuable insights into public attitudes toward antibiotic access and stewardship policies. The study’s strength lies in this innovative methodology and cross‑regional scope, capturing diverse perspectives shaped by local health systems and economic contexts.

  The decision to conduct Deliberative Polling® in six middle‑income countries across three southern‑hemisphere continents is particularly valuable. Although citizens in all settings share a common burden of bacterial infections, antibiotic use, and antibiotic resistance, the manuscript captures striking diversity in perspectives shaped by distinct structural, economic, environmental, and health‑system contexts. This diversity strengthens the contribution and highlights the importance of understanding how public attitudes align with emerging AMR policy priorities. I found the manuscript genuinely fascinating to read, and I would like to commend the authors and all involved for assembling such a rich and thoughtfully designed study. The manuscript I have no doubt will make a significant impact.

**Major comments**

**1. Strengthen the policy framing of regional differences**

 The manuscript identifies notable divergences between LATAM (especially Brazil) and African/Asian participants regarding support for informal antibiotic access and attitudes toward restricting antibiotic use in food production. These findings are both fascinating and compelling, but the discussion currently stops short of fully unpacking their policy implications.

 I encourage the authors to expand the discussion to more explicitly connect these patterns to the structural and economic contexts shaping them.

 For example:

- In many African and Asian settings, health systems have more limited access to trained clinicians, and reliance on informal or semi‑formal providers (e.g., pharmacists, medicine vendors, para‑veterinarians). This creates a pragmatic rationale for supporting expanded antibiotic access through these channels. This context is essential for interpreting why participants may prioritise access over regulation, particularly given the high burden of treatable bacterial infections. In settings with limited clinical access, stewardship models that incorporate informal providers (e.g., training, supervision, supply‑chain regulation) may be more feasible than restrictive approaches that assume strong formal health systems.
- In contrast, the relative resistance among Brazilian and Colombian participants to restrictions on antibiotics in the food chain may reflect the economic centrality of livestock production in these countries. Acknowledging the role of agricultural dependence, export markets, and political economy would help clarify why stewardship interventions in this domain may be perceived as economically threatening. In countries with large livestock sectors, policy pathways that emphasise economic incentives, innovation in animal health, or gradual transitions may be more acceptable than abrupt regulatory restrictions.

Even a brief articulation of these differentiated pathways would help readers understand how the study’s insights translate into actionable policy considerations.

**2. Consider briefly acknowledging climate‑related drivers of AMR**

 As a suggestion, the authors may wish to briefly acknowledge in the introduction the growing evidence that climate change is accelerating the spread and evolution of antimicrobial resistance. Even a short reference would help situate AMR within the broader landscape of global environmental and health pressures.

 The authors might also consider noting that environmental pressures—including climate‑driven changes in disease ecology, water systems, and agricultural practices—can influence public perceptions of AMR in different ways across countries. This is **not** to suggest that the countries included in this study lack environmental awareness; rather, it reflects that regions with different environmental narratives or policy priorities (for example, some Nordic countries with strong public engagement on environmental issues) may exhibit distinct patterns of concern or support for certain AMR‑related policies. A brief acknowledgement of this could help readers understand how perceptions might vary in settings not included in the present analysis.

**Minor Typo in number 17 of the** **45 policy proposals**

 17. Specific government **[t]** funding should go to healthcare facilities to ensure that all have access to clean water for hand hygiene

**Conclusion**

 This is a valuable and timely manuscript with strong empirical foundations. I would again like to commend the authors for producing such an engaging and insightful piece of work. The global burden of AMR makes it increasingly important to understand how the public perceives antibiotic access, stewardship, and regulatory interventions, and this study offers rich and nuanced insights into these dynamics.

 I also strongly agree with the authors’ statement that Deliberative Polling® is a powerful method for community engagement on the existential challenge of antimicrobial resistance. The manuscript clearly demonstrates the value of this approach in surfacing informed public perspectives across diverse contexts.

 For AMR policies to be adopted and implemented globally, it will be essential to understand the specific social, economic, cultural, and political drivers within each country. The study’s regional variations in support underscore that global policy frameworks cannot assume uniform public priorities; effective implementation will depend on tailoring strategies to national contexts while maintaining shared international goals.

 Therefore, to translate public support into effective global action on AMR, it will be imperative to broaden this work beyond the six middle‑income countries included here. A truly global evidence base—capturing perspectives from low‑, middle‑, and high‑income settings—will be essential for designing policies that are both internationally coherent and locally feasible. Expanding the study would also help illuminate the diverse national drivers that shape public priorities, ensuring that global commitments are grounded in the realities of the communities they aim to serve. I hope that future funding mechanisms can be mobilised to support this direction of research.

Is the work clearly and accurately presented and does it cite the current literature?

Yes

If applicable, is the statistical analysis and its interpretation appropriate?

Yes

Are all the source data underlying the results available to ensure full reproducibility?

Yes

Is the study design appropriate and is the work technically sound?

Yes

Are the conclusions drawn adequately supported by the results?

Yes

Are sufficient details of methods and analysis provided to allow replication by others?

Yes

Reviewer Expertise:

Antimicrobial resistance; Microbiome; host-microbial interactions; mucosal immunology; AMR Policy and Microbial-based product regulation.

I confirm that I have read this submission and believe that I have an appropriate level of expertise to confirm that it is of an acceptable scientific standard.

# Response

**Published:** 2026-03-23

**Authors:** Louise Gough

**Affiliations:** The Trinity Challenge, Cambridge, England, UK

Thank you for reviewing our manuscript and for your kind words.

**Point 1- In many African and Asian settings, health systems have more limited access to trained clinicians, and reliance on informal or semi** **‑formal providers (e.g., pharmacists, medicine vendors, para** **‑veterinarians). This creates a pragmatic rationale for supporting expanded antibiotic access through these channels. This context is essential for interpreting why participants may prioritise access over regulation, particularly given the high burden of treatable bacterial infections. In settings with limited clinical access, stewardship models that incorporate informal providers (e.g., training, supervision, supply** **‑chain regulation) may be more feasible than restrictive approaches that assume strong formal health**

**Response: **We absolutely agree with your point. We have tried to encompass it in the discussion as follows: Weaker health systems in Africa and Asia and challenges in access to health care workers leads to increased reliance on informal antibiotic sellers such as retail pharmacists, medicine vendors, and the allied veterinarian workforce for animals. This may explain the greater support for informal sellers to be able to increase access of citizens to antibiotics in African and Asian countries. Increased access to antibiotics could have a major impact on the close to 3 million people dying each year of bacterial sepsis due to antibiotic-sensitive bacterial infections.  

**Point 2- In contrast, the relative resistance among Brazilian and Colombian participants to restrictions on antibiotics in the food chain may reflect the economic centrality of livestock production in these countries. Acknowledging the role of agricultural dependence, export markets, and political economy would help clarify why stewardship interventions in this domain may be perceived as economically threatening. In countries with large livestock sectors, policy pathways that emphasise economic incentives, innovation in animal health, or gradual transitions may be more acceptable than abrupt regulatory restrictions.**

**Response: **Our current discussion does allude to this point, but we have added: ‘in relation to export markets and the political economy’

**Point 3- Consider briefly acknowledging climate‑related drivers of AMR** **As a suggestion, the authors may wish to briefly acknowledge in the introduction the growing evidence that climate change is accelerating the spread and evolution of antimicrobial resistance. Even a short reference would help situate AMR within the broader landscape of global environmental and health pressures. The authors might also consider noting that environmental pressures—including climate** **‑driven changes in disease ecology, water systems, and agricultural practices** **—can influence public perceptions of AMR in different ways across countries. This is not to suggest that the countries included in this study lack environmental awareness; rather, it reflects that regions with different environmental narratives or policy priorities (for example, some Nordic countries with strong public engagement on environmental issues) may exhibit distinct patterns of concern or support for certain AMR** **‑related policies. A brief acknowledgement of this could help readers understand how perceptions might vary in settings not included in the present analysis.**

**Response: **Thank you. We have added the following to the introduction: 'The effects of climate change on pathogens and their vectors is already accelerating bacterial resistance, through direct effects on bacteria themselves and by increasing the burden of infectious diseases in general e.g., increasing vector-borne infections by providing new ecologies for vectors. This in turn will increase transmission of pathogens to humans, resulting in increasing presentation of people with undifferentiated fevers to prescribers who will often inappropriately prescribe antibiotics'.  

**Point 4- Minor Typo in number 17 of the 45 policy proposals**

**Response: **Deleted. Thank you.
